# A CGA/EGFR/GATA2 positive feedback circuit confers chemoresistance in gastric cancer

**DOI:** 10.1172/JCI154074

**Published:** 2022-03-15

**Authors:** Tianyu Cao, Yuanyuan Lu, Qi Wang, Hongqiang Qin, Hongwei Li, Hao Guo, Minghui Ge, Sarah E. Glass, Bhuminder Singh, Wenyao Zhang, Jiaqiang Dong, Feng Du, Airong Qian, Ye Tian, Xin Wang, Cunxi Li, Kaichun Wu, Daiming Fan, Yongzhan Nie, Robert J. Coffey, Xiaodi Zhao

**Affiliations:** 1 State Key Laboratory of Cancer Biology, National Clinical Research Center for Digestive Diseases, Xijing Hospital of Digestive Diseases, Fourth Military Medical University, Xi’an, China.; 2 CAS Key Laboratory of Separation Science for Analytical Chemistry, Dalian Institute of Chemical Physics, Chinese Academy of Sciences, Dalian, China.; 3 State Key Laboratory of Translational Medicine and Innovative Drug Development, Jiangsu Simcere Diagnostics Co., Ltd., Nanjing, China.; 4 Department of Medicine, Vanderbilt University Medical Center, Nashville, Tennessee, USA.; 5 Key Laboratory for Space Biosciences and Biotechnology, School of Life Sciences, Northwestern Polytechnical University, Xi’an, China.; 6 Department of Gastroenterology, Tangdu Hospital, Fourth Military Medical University, Xi’an, China.; 7 Beijing Institute of Human Reproduction and Genetics Medicine, Beijing, China.; 8 Jiaen Genetics Laboratory, Beijing Jiaen Hospital, Beijing, China.

**Keywords:** Gastroenterology, Oncology, Gastric cancer, Oncogenes

## Abstract

De novo and acquired resistance are major impediments to the efficacy of conventional and targeted cancer therapy. In unselected gastric cancer (GC) patients with advanced disease, trials combining chemotherapy and an anti-EGFR monoclonal antibody have been largely unsuccessful. In an effort to identify biomarkers of resistance so as to better select patients for such trials, we screened the secretome of chemotherapy-treated human GC cell lines. We found that levels of CGA, the α-subunit of glycoprotein hormones, were markedly increased in the conditioned media of chemoresistant GC cells, and CGA immunoreactivity was enhanced in GC tissues that progressed on chemotherapy. CGA levels in plasma increased in GC patients who received chemotherapy, and this increase was correlated with reduced responsiveness to chemotherapy and poor survival. Mechanistically, secreted CGA was found to bind to EGFR and activate EGFR signaling, thereby conferring a survival advantage to GC cells. N-glycosylation of CGA at Asn52 and Asn78 is required for its stability, secretion, and interaction with EGFR. GATA2 was found to activate CGA transcription, whose increase, in turn, induced the expression and phosphorylation of GATA2 in an EGFR-dependent manner, forming a positive feedback circuit that was initiated by GATA2 autoregulation upon sublethal exposure to chemotherapy. Based on this circuit, combination strategies involving anti-EGFR therapies or targeting *CGA* with microRNAs (miR-708-3p and miR-761) restored chemotherapy sensitivity. These findings identify a clinically actionable CGA/EGFR/GATA2 circuit and highlight CGA as a predictive biomarker and therapeutic target in chemoresistant GC.

## Graphical abstract

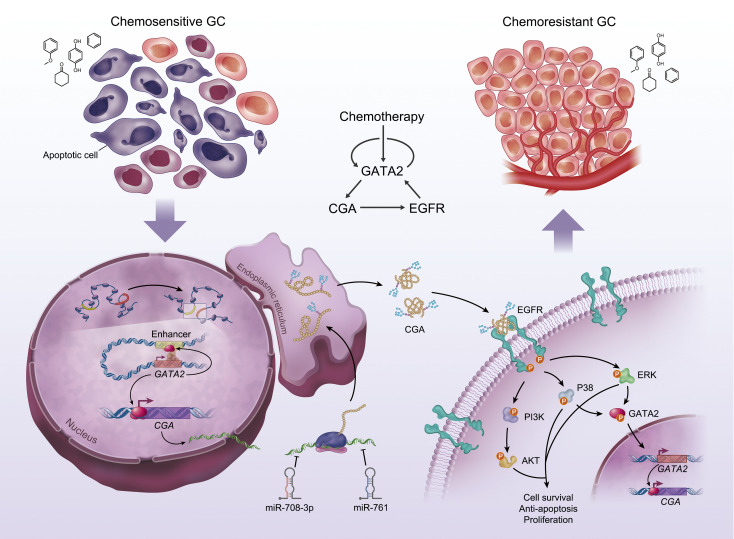

## Introduction

Gastric cancer (GC) is the fifth most common malignancy worldwide but is the third leading cause of cancer-related deaths (1). The high mortality of GC is mainly attributed to late diagnosis and limited treatment options (2). Although responses to chemotherapy have been reported in up to 60% of GC patients, most patients eventually develop chemoresistance and experience recurrence, with a 5-year survival rate of less than 10% (3, 4). Understanding how cancer cells overcome chemotherapy-induced cell death is critically important to improve patient survival. Emerging evidence reveals that factors secreted by cancer cells, including soluble proteins and insoluble vesicles, contribute to chemoresistance (5). Although it is estimated that nearly 30% of the transcripts in the stomach encode secreted proteins (6), little is known about the GC secretome and its alterations in response to chemotherapy.

Glycoproteins are abundant components in the secretome of both normal and tumor cells (5). An important subset of glycoproteins is the family of glycoprotein hormones, which includes human chorionic gonadotropin (hCG), follicle-stimulating hormone (FSH), luteinizing hormone (LH), and thyroid-stimulating hormone (TSH) (7). These heterodimeric proteins are composed of a common α-subunit (glycoprotein hormone α polypeptide, CGA) and 1 of 4 specific β-subunits (7). While it is known that the different β-subunits of each hormone confer receptor and biological specificity to play vital roles in reproduction, sexual development, and thyroid function (8), the function of unbound CGA is less clear. Structural studies have identified a characteristic cystine-knot motif in the central core of CGA, which shares high similarity with the structures of some growth factors such as NGF, TGF-β, and PDGF-β (9). Indeed, in vitro studies have demonstrated that CGA plays a role in the maintenance of anchorage-independent growth in lung and cervical tumor cells (10, 11). This observation has led investigators to propose a cell growth–related role for CGA, although the precise mechanism is not known.

Previous studies have shown that overexpression of EGFR promotes chemoresistance in cancer cells (12), and that EGFR-mediated activation of the MAPK and PI3K pathways can be observed following chemotherapy exposure (13, 14). However, the mechanism by which EGFR signaling is activated during chemoresistance is poorly understood. By acquiring dependence on a limited number of critical signaling pathways for survival, cancer cells may become sensitive to inhibition of these pathways, which has been the basis for targeted therapy (15). In line with this, enhanced sensitivity to anti-EGFR therapy has been reported in chemoresistant cells (16). Since EGFR is frequently overexpressed in GC and this overexpression correlates with poor prognosis (17), combining anti-EGFR therapy with chemotherapy provides a rationale to treat patients with chemoresistant GC. Indeed, several phase II clinical trials demonstrated a benefit of combining chemotherapy and anti-EGFR therapy in GC patients (18–20); however, randomized, open-label phase III trials failed to show a clinical benefit for anti-EGFR therapy in GC treatment (21, 22). Since the phase III trials were assessed in a molecularly unselected population, one possible factor contributing to this inconsistency is the lack of predictive biomarkers to identify patients who tend to develop EGFR-dependent tumor growth and drug resistance.

Here, we found that secreted CGA from chemoresistant GC cells confers and maintains resistance to chemotherapy. We also determined that N-glycosylated CGA bound to EGFR and activated EGFR signaling, which provided a survival advantage to cancer cells during chemotherapy. Lastly, we found that CGA was transcriptionally activated by GATA2 and uncovered a CGA/EGFR/GATA2 positive feedback circuit that was initiated by GATA2 autoregulation upon chemotherapy exposure. Our study provides evidence that CGA may serve as a plasma biomarker and a therapeutic target to treat chemoresistant GC.

## Results

### CGA is upregulated in chemoresistant GC cells and tissues.

**Figure 1:**
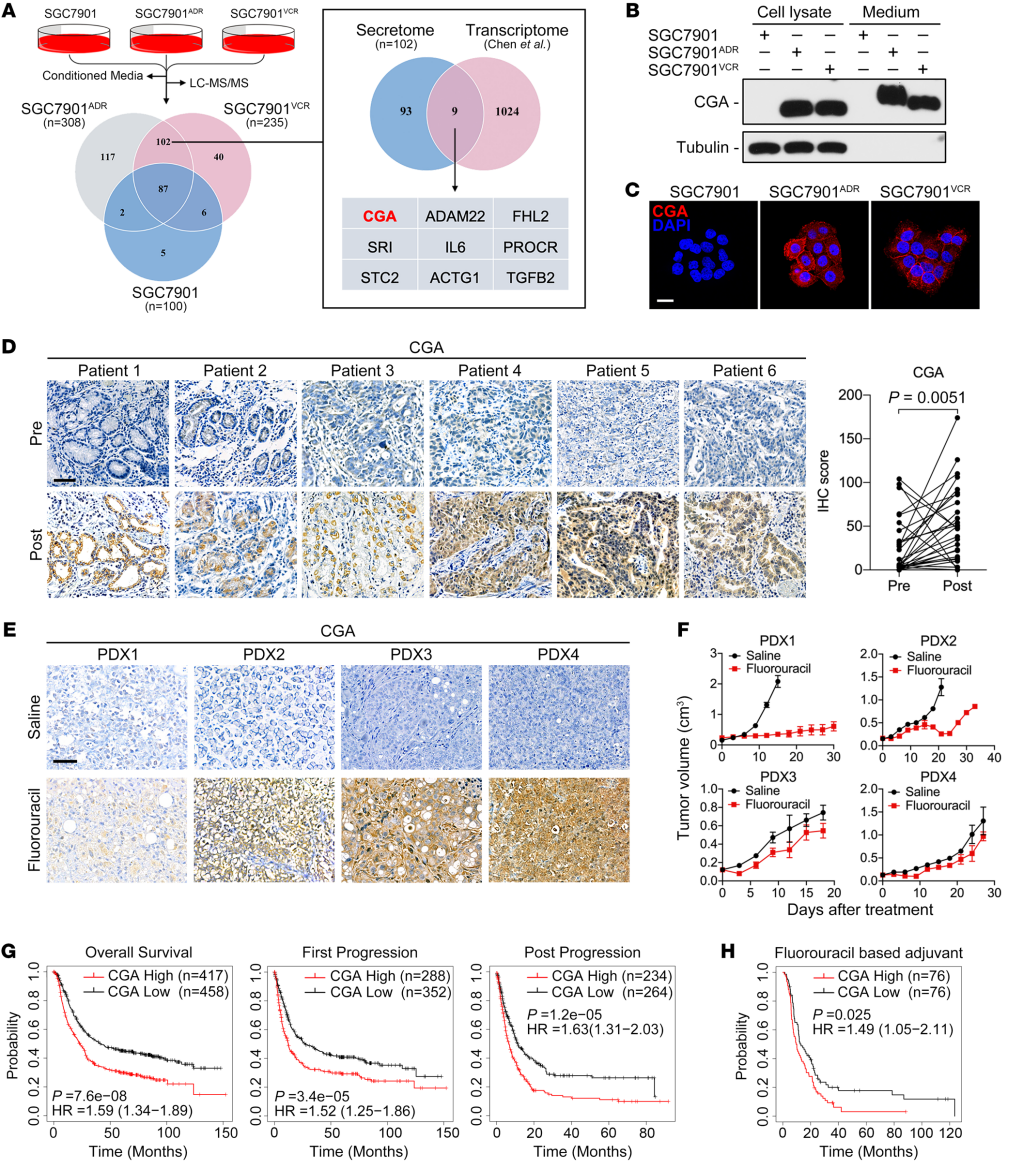
CGA is upregulated in chemoresistant GC cells and tissues. (**A**) Quantitative analysis comparing secretomes of SGC7901 and MDR cells. Venn diagram of the secreted proteins identified in indicated cells (left) and the overlap between upregulated genes in the secretome and transcriptome of MDR cells (right). (**B** and **C**) Immunoblotting (**B**) and representative IF images (**C**) of CGA in SGC7901 and MDR cells. Scale bar: 20 μm. (**D**) IHC staining of CGA in 6 representative nonresponsive human GC specimens (*n =* 31) obtained before and after chemotherapy. Scale bar: 50 μm. The IHC scores of CGA are shown. *P* value was calculated by Wilcoxon’s matched-pairs signed-rank test. (**E** and **F**) Mice with subcutaneous GC PDXs (*n =* 3–5) received indicated treatment every 3 days (fluorouracil, 60 mg/kg, i.p. injection). IHC staining of CGA in PDXs was performed (**E**) and corresponding tumor growth curves are shown (**F**). Data are presented as mean ± SEM. (**G** and **H**) Kaplan-Meier analyses of correlations between CGA expression and overall survival, first-progression or post-progression survival of GC patients (**G**) and between CGA expression and overall survival of GC patients who received fluorouracil-based adjuvant therapy (**H**) in the KM plotter database.

The multidrug-resistant (MDR) GC cell sublines SGC7901^ADR^ and SGC7901^VCR^ were previously established from the chemosensitive GC cell line SGC7901 in vitro by stepwise selection with broad-spectrum chemotherapeutic agents Adriamycin and vincristine, respectively (23). We validated that SGC7901^ADR^ and SGC7901^VCR^ cells exhibited varying degrees of cross-resistance to the first-line chemotherapeutic agents for GC, including fluorouracil, Adriamycin, and paclitaxel (Supplemental Figure 1A; supplemental material available online with this article; https://doi.org/10.1172/JCI154074DS1). To identify novel biomarkers for chemoresistance, we used liquid chromatography–tandem mass spectrometry (LC-MS/MS) to analyze proteins secreted by SGC7901^ADR^ and SGC7901^VCR^ cells and compared these data sets to proteins secreted by parental SGC7901 cells. A total of 100, 308, and 235 proteins were identified in the conditioned medium from SGC7901, SGC7901^ADR^, and SGC7901^VCR^ cells, respectively, with 102 proteins that were secreted exclusively from MDR cell lines (Figure 1A and Supplemental Table 1). Functional annotation analysis revealed compositional differences between the secretomes of SGC7901 and MDR cells (Supplemental Figure 1B). When the secretome data were cross-referenced to our previously reported gene expression profiling data (24), 9 genes were found to be upregulated at both the protein and mRNA level (Figure 1A). Among these candidates, *CGA* encodes the α-subunit of glycoprotein hormones (25). Similar to α-fetoprotein (26), CGA has been detected in pregnant women and patients with trophoblastic tumors (27), but its roles in chemoresistance are unknown. Therefore, we focused on *CGA* and investigated whether it could serve as a potential biomarker to predict GC chemoresistance or be involved in GC progression.

An increased expression of CGA was confirmed in lysates and media of MDR cells (Figure 1, B and C). To examine the relevance of CGA in GC chemoresistance, we collected 37 cases of paired biopsied or surgically resected specimens from GC patients before and after neoadjuvant chemotherapy (Supplemental Table 2). Among the patients who did not respond to chemotherapy, their posttreatment tumors exhibited increased focal or diffuse immunohistochemical (IHC) staining of CGA compared with their pretreatment samples (Figure 1D). However, we did not observe a substantial increase in CGA expression in tumors from patients who responded to chemotherapy (Supplemental Figure 1C). We further established subcutaneous GC patient–derived xenografts (PDXs) in mice (Supplemental Table 3). The PDXs exhibited variable changes in CGA expression after treatment with fluorouracil (Figure 1E), which is consistent with the heterogeneity observed in human GC samples. Notably, the tumors derived from PDXs with high portions of CGA-positive cells regrew shortly after chemotherapy (Figure 1F), suggesting a functional connection between CGA expression and the responsiveness of the tumor to chemotherapy. In addition, analyses using the Kaplan-Meier plotter (KM plotter) database (https://kmplot.com/analysis/) showed that a high *CGA* mRNA level was significantly correlated with a poor overall survival and first-progression or post-progression survival in GC patients (Figure 1G). In the fluorouracil-based adjuvant treatment arm, patients with high CGA–expressing tumors exhibited a shorter overall survival (Figure 1H). Collectively, these results indicate that CGA is pathologically and clinically associated with GC chemoresistance and patient outcome.

### CGA is important to maintain chemoresistance in GC cells.

**Figure 2:**
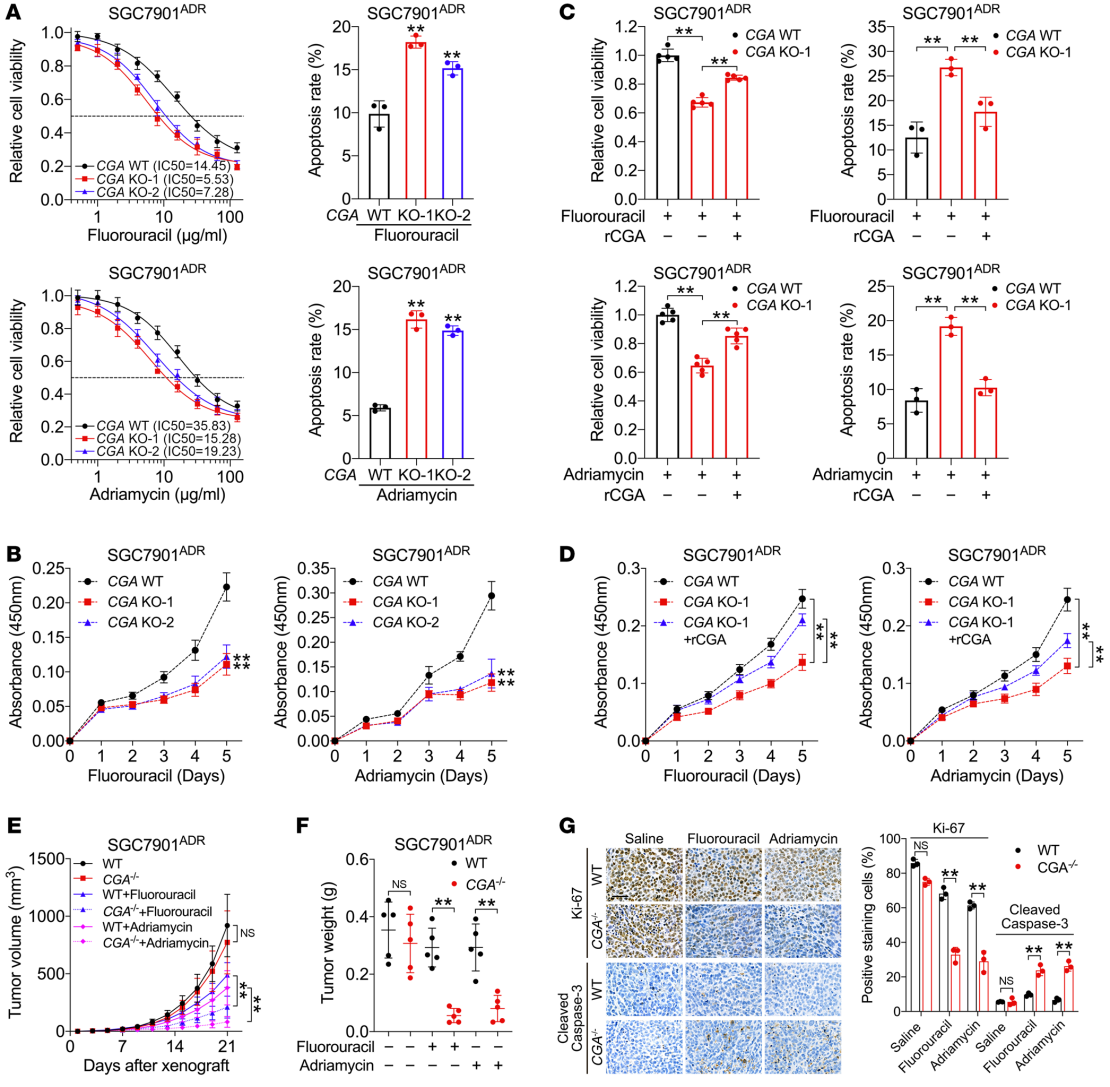
CGA is important to maintain chemoresistance in GC cells. (**A**) IC_50_ values and apoptosis of *CGA*-WT and -KO SGC7901^ADR^ cells treated with fluorouracil (10 μg/mL) or Adriamycin (10 μg/mL). (**B**) Growth curves of *CGA*-WT and -KO SGC7901^ADR^ cells treated with chemotherapy. (**C** and **D**) Viability, apoptosis (**C**), and growth curves (**D**) of *CGA*-WT and -KO SGC7901^ADR^ cells treated with or without rCGA (20 μg/mL) and chemotherapy. (**E**–**G**) *CGA*-WT and -KO SGC7901^ADR^ cells were injected subcutaneously into nude mice (*n =* 5). After tumors were palpable, mice received indicated treatment every 3 days (fluorouracil, 20 mg/kg, i.p. injection; Adriamycin, 8 mg/kg, i.p. injection). Tumor volume (**E**) and tumor weight (**F**) were measured. Ki-67 and cleaved caspase-3 staining and percentage in tumors is shown (**G**). Scale bar: 50 μm. Data are presented as mean ± SEM. **P < 0.01 by 1-way ANOVA with Dunnett’s multiple-comparison test (**A**–**D**), repeated-measures ANOVA test (**E**), or by Student’s *t* test (**F** and **G**).

To determine the roles of CGA in GC chemoresistance, we specifically deleted *CGA* (*CGA^–/–^*) by CRISPR/Cas9 genome editing in MDR cells (Supplemental Figure 2, A and B). Knockout of *CGA* did not cause an obvious change in cell proliferation in the absence of chemotherapy (Supplemental Figure 2C). Fluorouracil is one of the most widely used first-line chemotherapeutic agents for GC and was preferred for our in vivo and in vitro experiments; since SGC7901^ADR^ cells exhibit high resistance to Adriamycin and SGC7901^VCR^ cells exhibit high resistance to paclitaxel (Supplemental Figure 1A), the 2 cell sublines after *CGA* knockout were treated with the respective drug related to their resistance when 2 agents were validated. We found that *CGA^–/–^* SGC7901^ADR^ cells showed decreased therapeutic resistance and increased apoptosis in response to fluorouracil and Adriamycin (Figure 2A). Similar results were observed in *CGA^–/–^* SGC7901^VCR^ cells treated with fluorouracil and paclitaxel (Supplemental Figure 2D). *CGA* knockout also inhibited the proliferation of MDR cells exposed to chemotherapy (Figure 2B and Supplemental Figure 2E). Restoration of CGA by incubation with recombinant human CGA (rCGA) largely abrogated responsiveness to chemotherapy (Figure 2, C and D, and Supplemental Figure 2, F and G). CGA knockdown and subsequent rescue experiments in human GC cell lines BGC823 and MKN45, which exhibited relatively high levels of endogenous CGA, supported the role of CGA in chemoresistance (Supplemental Figure 3, A–C). To examine the effects of CGA in vivo, wild-type (WT) and *CGA^–/–^* MDR cells were engrafted subcutaneously into nude mice, followed by treatment with fluorouracil, Adriamycin, paclitaxel, or saline. We observed marked reductions in tumor volume and tumor weight in mice xenografted with *CGA^–/–^* tumors that received chemotherapy compared with mice with *CGA^–/–^* tumors that did not receive chemotherapy or mice with WT tumors receiving chemotherapy (Figure 2, E and F, and Supplemental Figure 3, D–F). *CGA^–/–^* tumors treated with chemotherapy contained fewer Ki-67–positive cells and more cleaved caspase-3–positive cells than the WT tumors (Figure 2G and Supplemental Figure 3G). These results indicate that CGA confers chemoresistance to GC cells and that targeting CGA sensitized GC cells to chemotherapy.

### CGA functions by binding to EGFR and activating EGFR signaling in GC cells.

Since secreted proteins usually function via paracrine and autocrine mechanisms (5), we first determined whether the effect of CGA on chemoresistance is dependent on its release from cells. We generated constructs that encode full-length (FL) CGA or a mutant with a truncated N-terminal signal peptide (ΔSP). Both constructs expressed intracellular CGA in *CGA^–/–^* cells, but the ΔSP mutation abolished secretion of CGA (Supplemental Figure 4A). Notably, transfection of FL CGA, but not the ΔSP mutant, restored the chemoresistance of *CGA^–/–^* cells (Supplemental Figure 4B), indicating that the chemoresistance-promoting effect of CGA is dependent on its secreted form. We next determined whether the activity of secreted CGA is dependent not only on CGA, the α-subunit itself, but also on 1 of the 4 β-subunits. Immunoblotting showed that the expression of the β-subunits (CGB, FSHB, LHB, and TSHB) was comparable in cell lysates but was barely detectable in the media from SGC7901 and MDR cells (Supplemental Figure 4C). Furthermore, knockdown of each β-subunit did not decrease the chemoresistance of MDR cells (Supplemental Figure 4, D and E), indicating that none of the β-subunits are necessary for CGA-mediated chemoresistance.

**Figure 3:**
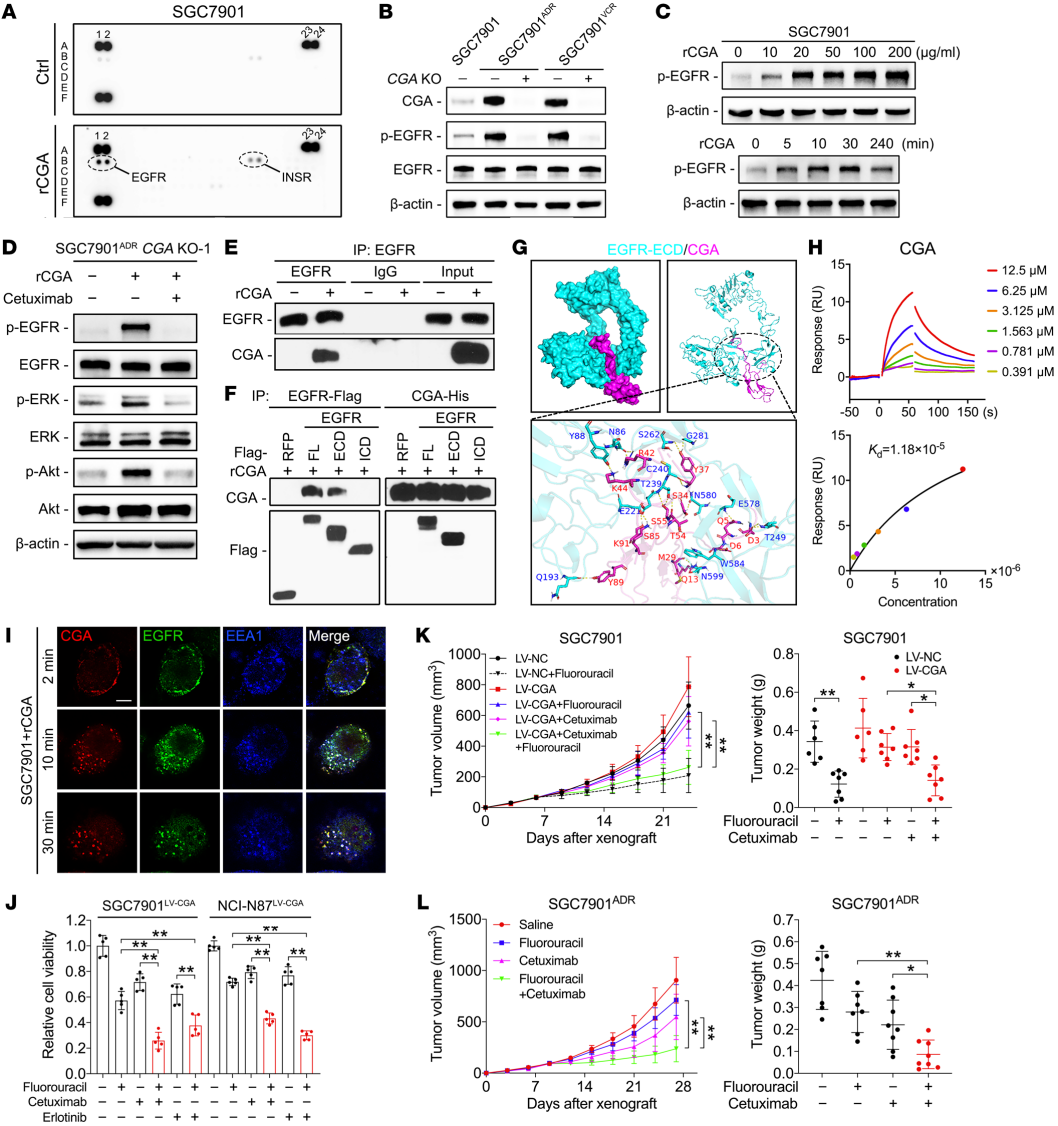
CGA functions by binding to EGFR and activating EGFR downstream signaling in GC cells. (**A**) Human phosphorylated RTK antibody array in SGC7901 cells serum starved for 12 hours and then treated with rCGA for 30 minutes. (**B**) Immunoblotting of CGA, EGFR, and p-EGFR in indicated serum-starved cells. (**C**) Immunoblotting of p-EGFR in serum-starved SGC7901 cells treated with indicated concentrations of rCGA (top) or treated with rCGA (20 μg/mL) at different time points (bottom). (**D**) Immunoblotting with indicated antibodies of lysates from *CGA^–/–^* SGC7901^ADR^ cells that were pretreated with cetuximab (10 μg/mL) followed by rCGA treatment. (**E**) Immunoblotting of lysates from SGC7901 cells that were incubated with rCGA and immunoprecipitated with anti-EGFR antibody or normal IgG. (**F**) Immunoblotting of lysates from SGC7901 cells transfected with Flag-tagged RFP or EGFR containing FL, ECD, or ICD plasmids, treated with purified His-tagged rCGA, and subjected to anti-Flag and anti-His immunoprecipitation. (**G**) Molecular docking analysis of CGA to the ECD of EGFR. (**H**) SPR analysis of the interaction between CGA and the ECD of EGFR. Raw response (RU) curves (top) from a representative experiment were fitted to a 1-site-specific kinetic model (bottom) to derive on and off rates and a *K*_d_ value for the interaction. (**I**) IF staining of CGA, EGFR, and early endosome marker EEA1 in SGC7901 cells treated with rCGA at 37°C for a 30-minute time course. Scale bar: 10 μm. (**J**) Viability of SGC7901 and NCI-N87 cells stably expressing CGA, treated with fluorouracil, cetuximab, erlotinib (20 nM), or their combination. (**K** and **L**) SGC7901 cells stably expressing CGA and control SGC7901 cells (**K**) or SGC7901^ADR^ cells (**L**) were injected subcutaneously into nude mice (*n =* 6–8). When the tumor size reached 100 mm^3^, mice received indicated treatment every 3 days (fluorouracil, 20 mg/kg, i.p. injection; cetuximab, 1 mg/mouse, i.p. injection). Tumor volume and tumor weight were measured. Data are presented as mean ± SEM. *P < 0.05; **P < 0.01 by 1-way ANOVA with Bonferroni’s post hoc test (**J**–**L**) or by repeated-measures ANOVA with Bonferroni’s post hoc test (**K** and **L**).

Activation of a glycoprotein hormone receptor requires the heterodimeric forms of the α- and β-subunits (28), suggesting that it is unlikely that unbound CGA induces chemoresistance by binding to these hormone receptors. Since CGA staining was observed at the cell membrane (Figure 1C), we asked whether CGA could bind and activate certain cell surface receptors. To test this hypothesis, we performed phosphorylated receptor tyrosine kinase (RTK) antibody array analyses and found that rCGA treatment activated EGFR, but not ERBB2–4, in SGC7901 cells (Figure 3A). Consistently, EGFR phosphorylation was almost completely inhibited in *CGA^–/–^* MDR cells (Figure 3B), and the addition of rCGA induced the phosphorylation of EGFR in a concentration- and time-dependent manner in SGC7901 and NCI-N87 cells (Figure 3C and Supplemental Figure 4F). CGA activated EGFR downstream signaling, as evidenced by phosphorylation of ERK and AKT in *CGA^–/–^* SGC7901^ADR^ and NCI-N87 cells; these effects were blocked by cetuximab, an EGFR-neutralizing monoclonal antibody that binds to the extracellular domain (ECD) of EGFR (Figure 3D and Supplemental Figure 4G). These observations prompted us to investigate whether CGA interacts with EGFR. Coimmunoprecipitation indicated that CGA could bind to EGFR in SGC7901 cells (Figure 3E). Reciprocal immunoprecipitation confirmed the interaction between CGA and the ECD of EGFR (Figure 3F and Supplemental Figure 4H). We then used the ClusPro server (29) to perform molecular docking simulations of CGA to the ECD of EGFR. The molecular visualization results showed that there were approximately 19 amino acid residues of CGA that were involved in binding to residues in the ECD of EGFR through hydrogen bond interactions (Figure 3G and Supplemental Table 4). Furthermore, CGA showed rapid association with the sensor chip–immobilized ECD of EGFR, producing a concentration-dependent resonance signal using standard surface plasmon resonance (SPR), with a calculated dissociation constant (*K*_d_) of 1.18 × 10^−5^ M (Figure 3H). Consistently, immunofluorescence (IF) demonstrated that rCGA colocalized with EGFR at the cell membrane and induced internalization of EGFR within the cytoplasm (Figure 3I and Supplemental Figure 4I). These results indicate that CGA binds to EGFR and activates EGFR signaling in GC cells.

To better understand the mechanism underlying CGA-mediated chemoresistance, RNA sequencing was performed in MDR cells after CGA knockdown. A total of 1,311 and 839 genes were found to be differentially expressed (fold change > 2 and *P* < 0.05) in SGC7901^ADR^ and SGC7901^VCR^ cells after CGA knockdown, respectively (Supplemental Figure 4J). KEGG pathway enrichment analysis showed that the most significantly de-enriched pathways in both MDR cell lines included “ERBB signaling pathway,” “ATP-binding cassette transporters,” and “pathways in cancer” (Supplemental Figure 4K). These results are consistent with our proposed roles for CGA in regulating EGFR signaling networks. Alterations in other signaling pathways may be involved in CGA-mediated chemoresistance, as evidenced by the activation of the insulin receptor (Figure 3A).

The above results prompted us to ask whether blockade of EGFR prevents CGA-mediated chemoresistance in GC cells. We established clones stably expressing CGA from chemosensitive SGC7901 and NCI-N87 cells (Supplemental Figure 5A). CGA overexpression conferred fluorouracil resistance in both cell lines (Supplemental Figure 5B). We observed an enhanced cytotoxic effect of fluorouracil in combination with cetuximab or the EGFR tyrosine kinase inhibitor erlotinib in CGA-overexpressing cells and MDR cells (Figure 3J and Supplemental Figure 5C). Moreover, in CGA-overexpressing SGC7901 and MDR xenograft models, administration of fluorouracil or cetuximab alone only moderately slowed tumor growth, whereas the combination of fluorouracil and cetuximab resulted in a remarkable tumor reduction (Figure 3, K and L, and Supplemental Figure 5, D and E). These results indicate that EGFR blockade sensitized CGA-mediated chemoresistant cells to chemotherapy, highlighting that anti-EGFR therapy may represent an effective strategy to treat GC with high CGA expression.

### N-linked glycosylation is required for CGA-induced chemoresistance.

**Figure 4:**
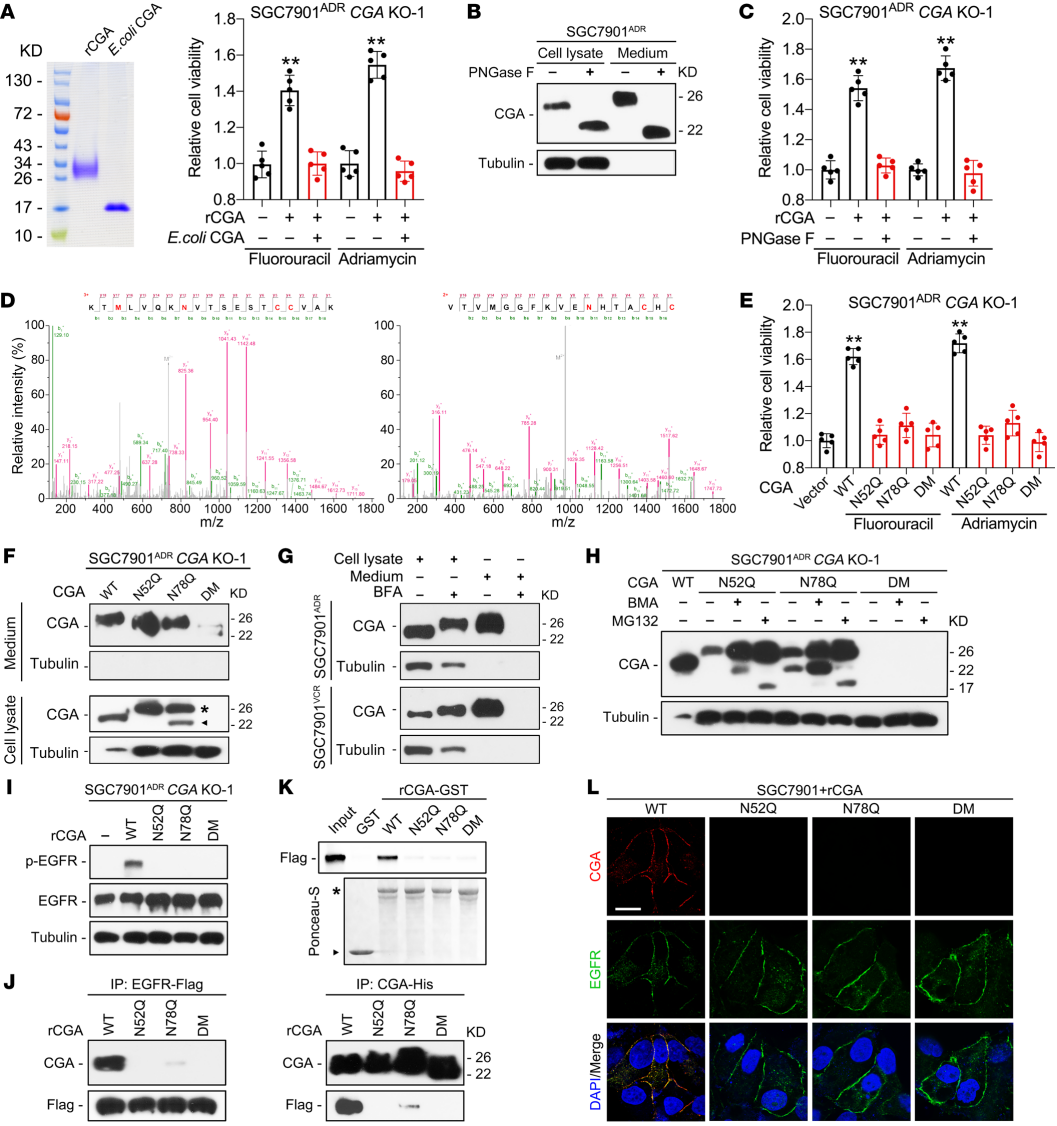
N-glycosylation is required for CGA-induced chemoresistance. (**A**) Left: SDS-PAGE of purified CGA from HEK293FT cells (rCGA) and *E*. *coli* (*E*. *coli* CGA). Right: Viability of *CGA^–/–^* SGC7901^ADR^ cells treated with rCGA or *E*. *coli* CGA and chemotherapy. (**B** and **C**) Immunoblotting of lysate and conditioned medium from PNGase F–treated SGC7901^ADR^ cells (**B**), with viability measured in indicated cells treated with chemotherapy (**C**). (**D**) MS/MS spectra of CGA secreted by SGC7901^ADR^ cells shows 2 N-glycosylation sites, Asn52 (left) and Asn78 (right), in CGA. N in red indicates the glycosylation sites. (**E**) Viability of *CGA^–/–^* SGC7901^ADR^ cells transfected with WT, N52Q, N78Q, or N52Q/N78Q double mutant (DM) CGA and treated with chemotherapy. (**F**) Immunoblotting of lysates and conditioned medium CGA from *CGA^–/–^* SGC7901^ADR^ cells transfected with WT, N52Q, N78Q, or DM CGA. Asterisk and arrowhead indicate CGA band shifts. (**G**) Immunoblotting of lysate and conditioned medium CGA from MDR cells treated with BFA (5 nM). (**H**) Immunoblotting of CGA from *CGA^–/–^* SGC7901^ADR^ cells transfected with WT, N52Q, N78Q, or DM CGA and treated with BMA (1 μM) or MG132 (10 μM). (**I**) Immunoblotting of p-EGFR and EGFR in *CGA^–/–^* SGC7901^ADR^ cells treated with purified WT, N52Q, N78Q, or DM rCGA. (**J**) Immunoblotting of lysates from SGC7901 cells transfected with Flag-tagged EGFR were incubated with purified His-tagged CGA after immunoprecipitation with anti-Flag and anti-His antibodies. (**K**) Top: Immunoblotting for Flag of bound proteins after GST or GST fusion proteins were incubated with equal amounts of lysates from Flag-tagged EGFR-ECD–expressing HEK293T cells. Bottom: Ponceau-S staining to detect bait proteins. Arrowhead and asterisk indicate GST and GST fusion proteins, respectively. (**L**) IF staining of CGA, EGFR, and DAPI staining in SGC7901 cells treated with WT, N52Q, N78Q, or DM rCGA for 10 minutes at 4°C. Scale bar: 10 μm. Data are presented as mean ± SEM. **P < 0.01 by 1-way ANOVA with Dunnett’s multiple-comparison test (**A**, **C**, and **E**).

Glycosylation influences the structure and biological function of substrate proteins (30). CGA contains 2 N-linked glycosylation (N-glycosylation) sites (25), but their functional importance in chemoresistance has not been determined. We purified CGA from *Escherichia coli* in which proteins are not glycosylated and found that bacterially expressed CGA failed to induce chemoresistance (Figure 4A). Incubation with rCGA pretreated with PNGase F, which removes N-linked oligosaccharides from glycoproteins, was unable to restore chemoresistance in *CGA^–/–^* MDR cells (Figure 4, B and C), indicating that N-glycosylation is required for CGA-mediated chemoresistance. LC-MS/MS analysis confirmed that CGA secreted from SGC7901^ADR^ cells was modified by N-glycosylation at Asn52 and Asn78 (Figure 4D). We also found that reconstituting *CGA^–/–^* MDR cells with CGA constructs containing mutations in the glycosylation sites could not restore chemoresistance (Figure 4E).

The inability of N-glycosylation mutants to induce chemoresistance may be due to alterations in expression, protein stability, or secretion of CGA. Mutations did not affect CGA mRNA levels (Supplemental Figure 6A) but reduced both intracellular and secreted CGA protein levels (Figure 4F and Supplemental Figure 6B), which was consistent with previously reported findings (31). A nonglycosylated protein should exhibit a lower molecular weight (MW) than its glycosylated form, but instead we found an additional CGA protein band of a higher MW in N52Q and N78Q mutant–transfected cells (Figure 4F, asterisk). We noticed that this band was at the same MW as secreted CGA (Supplemental Figure 6C). Since the endoplasmic reticulum (ER) has quality control mechanisms to retain proteins with aberrant conformations (32), we speculated that an N-glycosylation mutation might impede CGA secretion. We treated MDR cells with brefeldin A (BFA), an ER-Golgi protein-trafficking inhibitor, and found that BFA treatment increased intracellular CGA (Figure 4G), suggesting that N-glycosylation is a quality control checkpoint for CGA. Moreover, we observed a faint band that was at a lower MW in N78Q mutant–transfected cells (Figure 4F, arrowhead), and this band was barely detectable when cells were transfected with the N52Q or N52Q/N78Q double mutant (DM) construct, prompting us to hypothesize that N-glycosylation mutations may also lead to enhanced degradation of CGA. Glycoproteins can be degraded by ER-associated degradation or autophagy pathways (33). We treated *CGA^–/–^* cells with bafilomycin A1 (BMA) and MG132, inhibitors of lysosomal and proteasomal proteolysis, respectively. The expression of WT CGA was enhanced when the cells were treated with BMA but not MG132, whereas expression of the N52Q and N78Q mutants increased not only by treatment with BMA but even more with MG132 (Figure 4H and Supplemental Figure 6D), suggesting that lysosomal degradation is the major pathway for WT CGA degradation, but N-glycosylation mutations shift the pathway to proteasomal degradation.

Since glycosylation can alter protein structure (30), we further investigated whether N-glycosylation mutations affect the interaction between CGA and EGFR. Notably, rCGA with N-glycosylation mutations failed to induce EGFR phosphorylation (Figure 4I and Supplemental Figure 6E). Furthermore, incubation of EGFR with WT or mutant rCGA revealed that the N52Q and N78Q mutations substantially limited the ability of CGA to bind EGFR (Figure 4J and Supplemental Figure 6F). Moreover, we validated the differences in interaction between the WT or mutant rCGA and the ECD of EGFR using a glutathione *S*-transferase (GST) fusion pull-down assay. The WT and mutant CGA-GST fusion proteins were purified from HEK239FT cells and then incubated with cell lysates from HEK293T cells transfected with the vector encoding the Flag-tagged ECD of EGFR. WT CGA-GST, but not GST, N52Q CGA-GST, N78Q CGA-GST, or DM CGA-GST fusion protein, was able to pull down the ECD of EGFR (Figure 4K). IF staining also showed that the mutant rCGA did not colocalize with EGFR at the cell surface (Figure 4L). These results indicate that N-glycosylation is required for CGA stability, secretion, and binding to EGFR.

### The reciprocal positive regulation between GATA2 and CGA/EGFR signaling.

**Figure 5:**
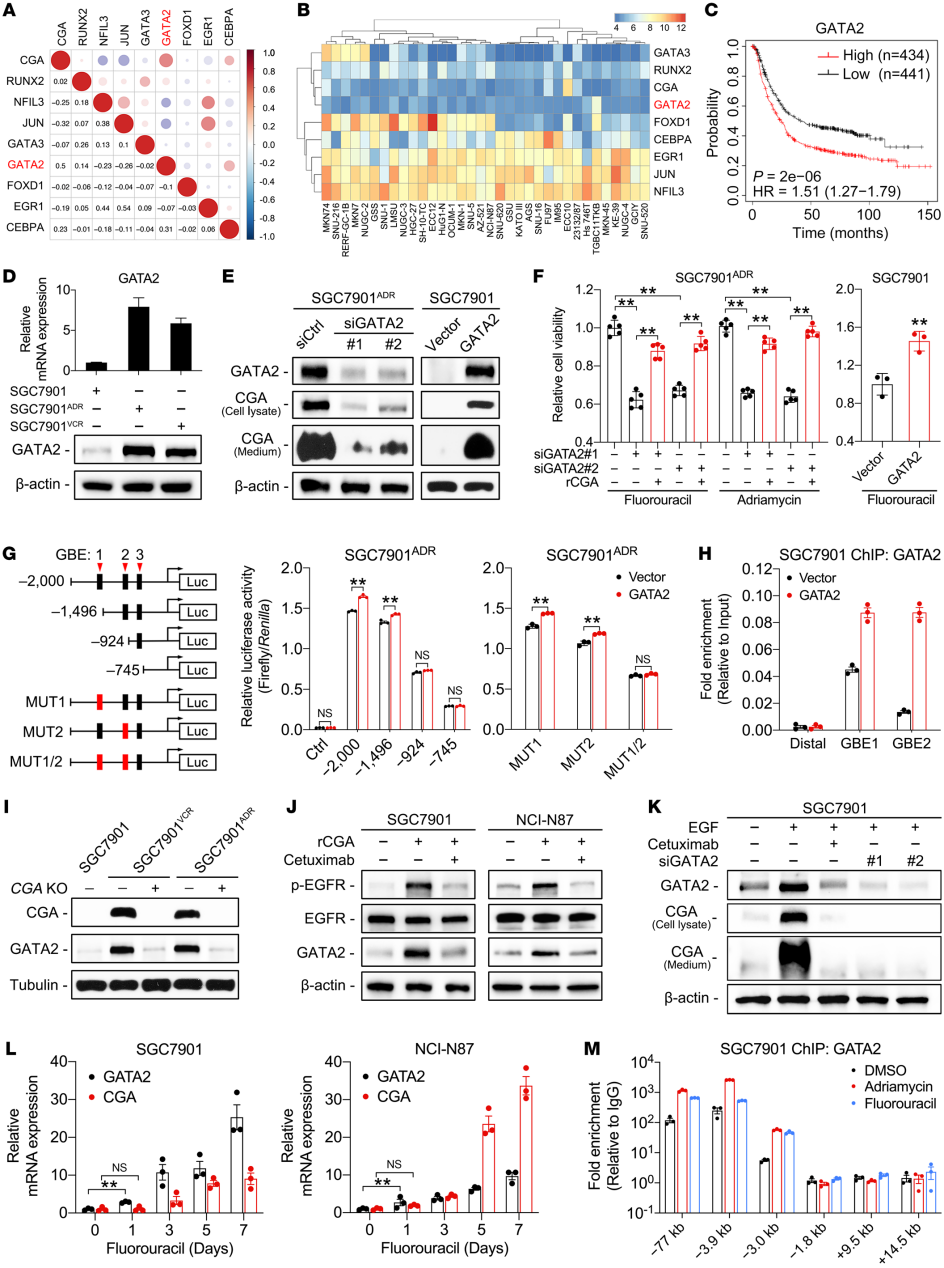
Reciprocal positive regulation between GATA2 and CGA/EGFR signaling. (**A** and **B**) Correlations between CGA and TFs in KM plotter (**A**) and CCLE (**B**) databases. Data evaluated using Pearson’s correlation coefficient. (**C**) Kaplan-Meier analysis of correlation between GATA2 expression and overall survival of GC patients using the KM plotter database. (**D**) RT-qPCR and immunoblotting of GATA2 in MDR and SGC7901 cells. (**E** and **F**) Immunoblotting of GATA2 and CGA in MDR cells transfected with 2 independent siRNAs against GATA2 (siGATA2) or a control siRNA (siCtrl) and in SGC7901 cells transfected with a GATA2 expression vector or empty vector (**E**). Viability was measured in the indicated cells treated with chemotherapy (**F**). (**G**) Left: Diagram of consecutive deletion and mutation constructs spanning the *CGA* promoter. GBE mutations shown in red boxes. Right: Luciferase reporter driven by the WT, deletion, or mutant (MUT) promoter was transfected into SGC7901^ADR^ cells. Luciferase activity was measured with or without GATA2 cotransfection. (**H**) ChIP with anti-GATA2 antibody in SGC7901 cells with or without GATA2 transfection. (**I**–**K**) Immunoblotting of CGA, GATA2, EGFR, and p-EGFR in indicated cells. (**L**) GATA2 and CGA expression in SGC7901 and NCI-N87 cells treated with low concentrations of fluorouracil (1 μg/mL) for the indicated times. (**M**) ChIP with anti-GATA2 antibody in SGC7901 cells treated with low-concentration chemotherapy. Data are presented as mean ± SEM. **P < 0.01 by 1-way ANOVA with Bonferroni’s post hoc test (**F**) or by Student’s *t* test (**G** and **L**).

Since the upregulation of CGA in MDR cells was detected at the mRNA level, we sought to explore the transcriptional regulation of CGA. Possible transcription factor (TF) binding motifs in the 2-kb promoter region of *CGA* were mapped in silico using the JASPAR database (https://jaspar.genereg.net/) and cross-referenced with our previous gene expression profiling data (24). We identified 8 TFs that were significantly increased in MDR cells (Supplemental Table 5). The correlations between CGA and these TFs in the KM plotter and the Cancer Cell Line Encyclopedia (CCLE) databases were analyzed; GATA2 showed the strongest positive correlation with CGA expression (Figure 5, A and B). High expression of GATA2 was associated with poor prognosis of GC patients and exhibited the highest hazard ratio among the 8 TFs (Figure 5C and Supplemental Figure 7A). These results suggest that GATA2 is a biologically and therapeutically important regulator of CGA, and we therefore selected it for further investigation.

The upregulation of GATA2 was validated in MDR cells (Figure 5D). Knockdown of GATA2 decreased CGA expression and attenuated chemoresistance, while addition of rCGA overcame the effect of GATA2 knockdown (Figure 5, E and F, and Supplemental Figure 7B). Overexpression of GATA2 in SGC7901 and NCI-N87 cells induced CGA expression and enhanced chemoresistance (Figure 5, E and F, and Supplemental Figure 7, C and D). By analyzing the *CGA* promoter region, we identified 3 putative GATA2-binding elements (GBEs) that are conserved across multiple species (Supplemental Figure 7E). Reporter constructs containing sequential deletions of the GBEs were transduced into MDR and HEK293T cells, and the results revealed that GBE1 and GBE2 are the major sites of GATA2 regulation for transcriptional activity of *CGA* (Figure 5G and Supplemental Figure 7F). Chromatin occupancy of GATA2 at GBE1 and GBE2 was further confirmed by chromatin immunoprecipitation (ChIP) and electrophoretic mobility shift assays (EMSAs) (Figure 5H and Supplemental Figure 7G). These results indicate that GATA2 regulates CGA transcription in GC cells.

Interestingly, we observed that GATA2 was largely decreased in *CGA^–/–^* MDR cells and rCGA treatment could induce GATA2 expression in SGC7901 and NCI-N87 cells (Figure 5, I and J). These phenomena suggest that a mutual regulatory mechanism exists between CGA and GATA2. Notably, cetuximab could block the CGA-induced GATA2 upregulation (Figure 5J), suggesting that CGA promotes GATA2 expression in an EGFR-dependent manner. Consistently, EGF treatment increased GATA2 and CGA levels, which were abrogated by cetuximab or GATA2 knockdown (Figure 5K and Supplemental Figure 8A). These results indicate that EGFR is involved in the reciprocal regulation between CGA and GATA2. We then determined which EGFR downstream signaling pathway(s) regulate GATA2 expression. We treated MDR cells with kinase, inhibitors including PD98059 (MEK/ERK inhibitor), SB202190 (p38 MAPK inhibitor), SP600125 (JNK inhibitor), and LY294002 (PI3K/AKT inhibitor), and found that SB202190 and PD98059 substantially reduced the expression of GATA2 and CGA (Supplemental Figure 8, B and C). SB203580 and PD98059 also suppressed phosphorylation of GATA2 at Ser192 (Supplemental Figure 8C), which is critical to the transcriptional activity of GATA2 (34). Functionally, combined inhibition of p38 and/or ERK signaling significantly diminished chemoresistance in MDR cells (Supplemental Figure 8D). Collectively, these results suggest a CGA/EGFR/GATA2 positive feedback circuit: GATA2 activates CGA transcription, and CGA increases EGFR activation, which in turn promotes expression and phosphorylation of GATA2 and GATA2-mediated CGA transcription.

To dissect how the CGA/EGFR/GATA2 circuit occurs under chemotherapy stress, we treated SGC7901 and NCI-N87 cells with a sublethal dose of fluorouracil or Adriamycin, which maintains the cell mortality rate at approximately 50%, allowing for the remaining cells to survive under continuous drug administration. We found that *GATA2* mRNA levels in the surviving cells continued to increase 1 day after treatment, while the increase in *CGA* mRNA was detected on the third day of treatment (Figure 5L and Supplemental Figure 8E), indicating that GATA2 expression was induced by chemotherapy and might be the driving factor in the CGA/EGFR/GATA2 circuit. Previous studies revealed that GATA2 undergoes positive transcriptional autoregulation during stem cell differentiation (35). Given that apoptosis-resistant cancer cells acquire stem cell–like properties (36), we tested whether chemotherapy induces the autoregulation of GATA2. As expected, chemotherapy substantially enhanced the occupancy of GATA2 on the GATA-binding motifs at –77 kb, –3.9 kb, and –3.0 kb in the *GATA2* locus in SGC7901 cells (Figure 5M). These results indicate that GATA2 is induced by chemotherapy and may function as a critical driver in the development of chemoresistance in GC cells.

### Elevated CGA and GATA2 levels in GC patients after chemotherapy.

**Figure 6:**
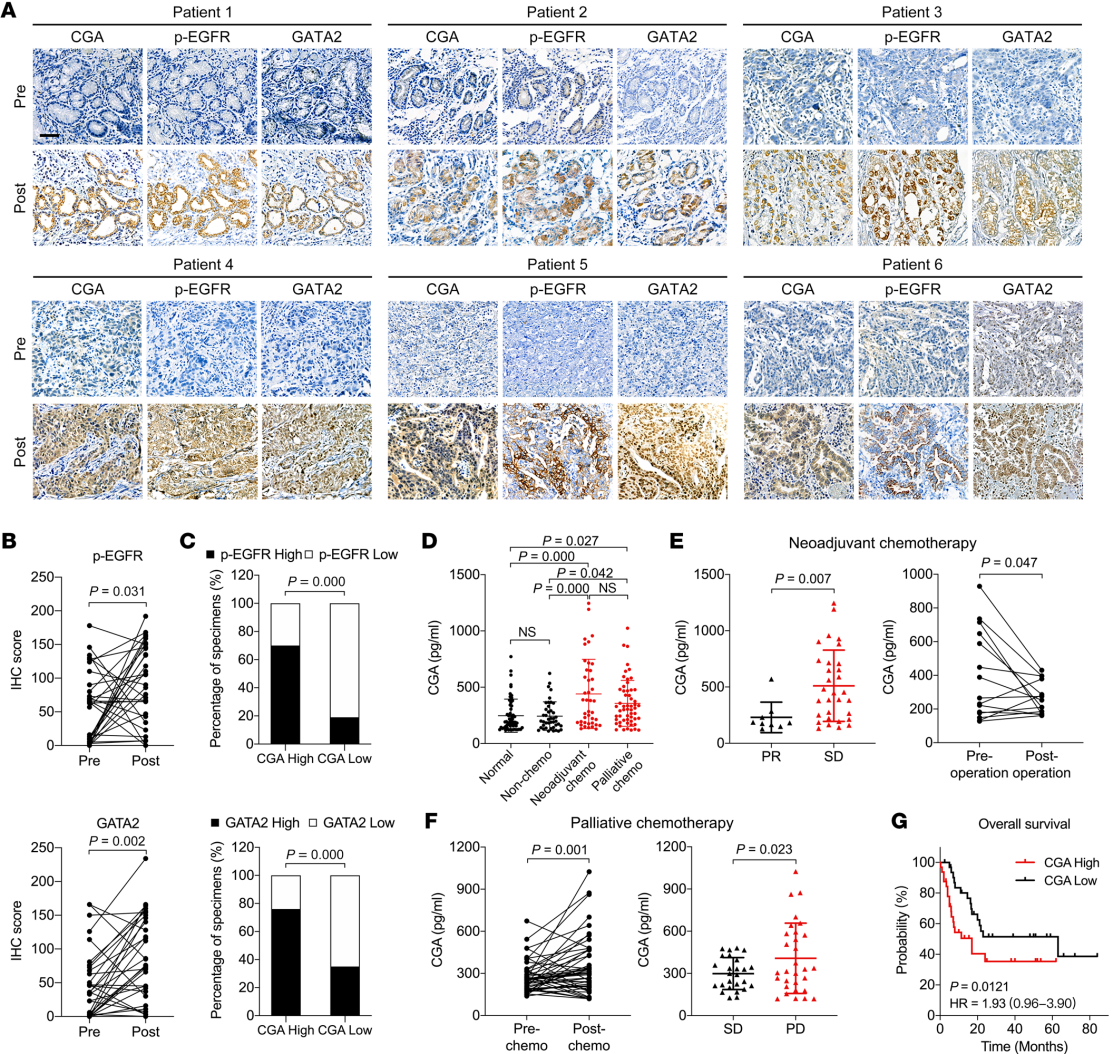
Elevated CGA and GATA2 expression levels in GC patients after chemotherapy. (**A** and **B**) IHC staining of CGA, p-EGFR, and GATA2 in 6 representative nonresponsive human GC specimens (*n =* 31) obtained before and after chemotherapy (**A**). Scale bar: 50 μm. IHC scores of p-EGFR and GATA2 are shown (**B**). The CGA images are the same as shown in Figure 1D. (**C**) Association between CGA and p-EGFR or GATA2 levels in nonresponsive GC specimens (*n =* 31) obtained after chemotherapy. (**D**) ELISA of CGA levels in plasma samples from healthy donors (normal, *n =* 57), newly diagnosed GC patients (non-chemo, *n =* 42), and neoadjuvant (*n =* 41) or palliative (*n =* 56) chemotherapy–treated GC patients. (**E**) Left: ELISA of plasma CGA from GC patients who received neoadjuvant chemotherapy with a partial response (PR, *n =* 9) or stable disease (SD, *n =* 31) status. Right: ELISA of plasma CGA from post- and preoperative samples of GC patients (*n =* 15) who received neoadjuvant chemotherapy. (**F**) Left: ELISA of plasma CGA from GC patients (*n =* 46) before and after palliative chemotherapy. Right: ELISA of plasma CGA from GC patients who received palliative chemotherapy and had progressive disease (PD, *n =* 30) or SD (*n =* 26) status. (**G**) Log-rank test for overall survival of GC patients (*n =* 64) with different CGA levels after neoadjuvant or palliative chemotherapy. Data are presented as mean ± SEM. *P* value was calculated by Wilcoxon’s matched-pairs signed-rank test (**B**), by χ^2^ test (**C**), by 1-way ANOVA with Bonferroni’s post hoc test (**D**), or by Student’s *t* test (**E** and **F**).

To examine whether the CGA/EGFR/GATA2 circuit occurs in GC patients, we stained for GATA2 and p-EGFR in 31 paired tumor specimens from patients who did not respond to chemotherapy. Consistent with the expression pattern of CGA, GATA2 staining increased in a focal or diffuse pattern in the tumors after chemotherapy (Figure 6, A and B). The level of p-EGFR was also high in the CGA-positive tumors after chemotherapy and in areas of CGA expression (Figure 6, A and B). Positive correlations between CGA and p-EGFR or GATA2 were observed (Figure 6C). Likewise, increased expression of GATA2 and p-EGFR was observed in the PDX tumors that regrew shortly after chemotherapy (Supplemental Figure 9A). Comparison of mRNA levels in a GC data set from the NCBI’s Gene Expression Omnibus (GEO) also revealed positive correlations among *CGA*, *EGFR*, and *GATA2*; intriguingly, positive correlations also were observed in a similar analysis of the GEO colorectal cancer (CRC) data set (Supplemental Figure 9, B and C). These data support the presence of CGA/EGFR/GATA2 circuit activity in GC patients.

Since secreted proteins from cancer cells constitute a rich source of biomarkers (5), we asked whether CGA in plasma could reflect the responsiveness of GC patients to chemotherapy. We measured CGA levels in blood samples from 57 healthy donors, 42 newly diagnosed GC patients before receiving treatment, and 97 GC patients who received neoadjuvant or palliative chemotherapy (Supplemental Tables 6–8). Enzyme-linked immunosorbent assay (ELISA) indicated a significant increase in plasma CGA levels in patients who received chemotherapy compared with those who did not receive prior therapy or healthy donors; however, the mean concentrations of plasma CGA between GC patients without chemotherapy and healthy donors were not significantly different (Figure 6D). Among the patients who received neoadjuvant chemotherapy, the plasma CGA levels in patients with stable disease were higher than in patients with partial response, and a decrease in CGA levels was observed in the postoperative plasma samples compared with their matched preoperative samples (Figure 6E). Notably, 32 (69.6%) of the 46 patients who received palliative chemotherapy showed higher plasma CGA levels after chemotherapy than their matched samples collected before chemotherapy, and the plasma CGA levels in those patients with progressive disease were higher than in patients with stable disease (Figure 6F). Furthermore, we used the median CGA plasma concentration (304.7 pg/mL) as a cutoff to divide the patients who received chemotherapy and survival follow-up survey into high- and low-CGA groups and found that a high level of CGA was associated with shorter survival (Figure 6G). These results suggest that plasma CGA levels might serve as a potential biomarker to reflect the responsiveness to chemotherapy and to predict survival in GC patients.

### miR-708-3p and miR-761 sensitize chemoresistant GC cells by targeting CGA.

**Figure 7:**
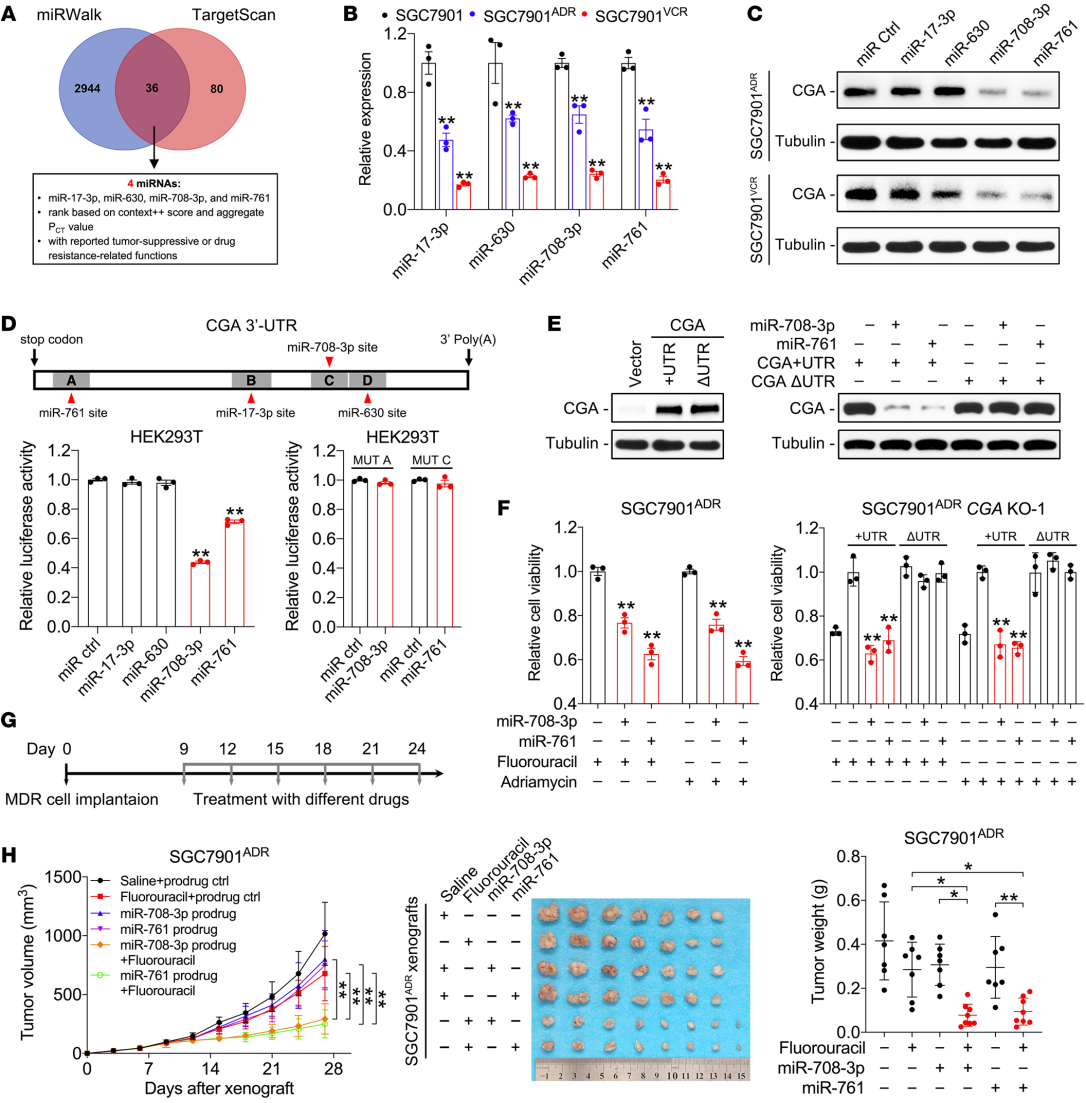
miR-708-3p and miR-761 sensitize chemoresistant GC cells by targeting CGA. (**A**) Diagram of screening for CGA-targeting miRNAs. Details can be found in Supplemental Table 9. (**B**) Expression of CGA-targeting miRNAs in SGC7901 and MDR cells. (**C**) Immunoblotting of CGA in MDR cells transfected with indicated miRNA mimics. (**D**) Top: Diagram of the predicted binding sites between indicated miRNAs and CGA 3′-UTR. Bottom: Luciferase activity derived from the CGA 3′-UTR reporter construct after cotransfection with indicated miRNA mimics. (**E**) Immunoblotting of CGA in *CGA^–/–^* SGC7901^ADR^ cells transfected with indicated constructs and/or miRNA mimics. (**F**) Viability of *CGA*-WT and -KO SGC7901^ADR^ cells transfected with indicated constructs and/or miRNA mimics and treated with chemotherapy. (**G** and **H**) Nude mice (*n =* 7–8) were implanted subcutaneously with SGC7901^ADR^ cells. When the tumor size reached 100 mm^3^, mice received indicated treatment every 3 days (**G**; fluorouracil, 20 mg/kg, i.p. injection; miRNA prodrugs, 1 nmol/mouse intratumoral injection). Tumor volume and tumor weight were measured (**H**). Data are presented as mean ± SEM. *P < 0.05; **P < 0.01 by 1-way ANOVA followed by Dunnett’s multiple-comparison test (**B**, **D**, and **F**) or by 1-way or repeated-measures ANOVA with Bonferroni’s post hoc test (**H**).

MicroRNAs (miRNAs) are negative regulators of gene expression and can be used as therapeutic agents (37). To identify possible miRNAs that target CGA, miRNAs recognizing putative binding sites on the 3′-UTR of CGA were predicted by multiple algorithms (Figure 7A). We found 36 candidates that target CGA (Supplemental Table 9), 4 of which (miR-17-3p, miR-630, miR-708-3p, and miR-761) have been reported to be involved in chemoresistance (38–41) and were selected for further analyses. Reduced expression of the 4 miRNAs was validated in MDR cells (Figure 7B). Immunoblotting showed that only miR-708-3p and miR-761 inhibited CGA expression in MDR cells (Figure 7C). miR-708-3p and miR-761 suppressed luciferase activity of the CGA 3′-UTR reporter in HEK293T cells, and this inhibitory effect was abolished when the binding sites were mutated (Figure 7D and Supplemental Figure 10A). miR-708-3p and miR-761 sensitized MDR cells to chemotherapy, whereas cotransfection with the CGA construct without the 3′-UTR, but not the construct containing the 3′-UTR, overcame the miRNA-mediated CGA repression and restored chemoresistance in *CGA^–/–^* MDR cells (Figure 7, E and F, and Supplemental Figure 10, B–D). These results indicate that CGA is a direct and functional target of miR-708-3p and miR-761 in GC cells.

To determine the roles of miR-708-3p and miR-761 in vivo, we synthesized chimeric miR-708-3p and miR-761 agents (termed miR-708-3p and miR-761 prodrugs hereafter) by using a Sephadex aptamer-tagged methionyl-tRNA scaffold-based method that enables miRNAs to capture cellular mRNA with natural characteristics (42). MDR cells were inoculated into nude mice, followed by treatment with chemotherapy and/or miRNA prodrugs (Figure 7G). Chemotherapy or intratumoral injection of the miR-708-3p or miR-761 prodrug modestly reduced tumor growth, whereas tumor shrinkage was more pronounced when the mice were injected with the combination of fluorouracil and miRNA prodrugs (Figure 7H and Supplemental Figure 10E). The miRNA prodrugs also increased apoptotic gene expression in response to chemotherapy (Supplemental Figure 10F). The mice treated with the miRNA prodrugs showed neither weight loss nor abnormalities in the liver and kidney (Supplemental Figure 10, G and H). These results suggest that miRNAs targeting CGA render GC cells more sensitive to chemotherapy and represent a potential therapeutic strategy to treat chemoresistance.

## Discussion

Cancer cells develop chemoresistance through intrinsic and/or acquired genetic modifications, as well as by nongenetic rewiring of signaling pathways that are crucial for cell survival (43). Emerging evidence has established that, in addition to intracellular processes, the cancer cell secretome also contributes to the development of chemoresistance (5). In the present study, we compared the secretomes of chemoresistant and chemosensitive GC cells and identified CGA as a potent “inducer” of chemoresistance that functions through activation of EGFR signaling, which is dependent on its N-glycosylation but not its β-subunit.

Chemoresistance is usually monitored through radiological assessment in clinical practice (44). Biomarkers that can predict the occurrence of chemoresistant or relapsed tumors are rare (45), especially in GC. Early studies found that expression of members of the interleukin (IL) family of cytokines, such as IL-6 and IL-8, was strongly correlated with cancer relapse and a poor response to chemotherapy (46, 47). Recent evidence indicates that chemotherapy exposure could alter the composition and abundance of the cancer cell secretome (5). Our work suggests that CGA might serve as a secreted biomarker as well as a therapeutic target in GC chemoresistance. Elevated CGA expression was observed in chemoresistant GC cells and PDXs, along with the blood and tissue samples from GC patients at the time of progression on chemotherapy. In addition, CGA may act as a positive predictor for using EGFR blockade. Since CGA functions by activating EGFR signaling, EGFR blockade may be indicated in GC patients with high CGA levels and a WT *KRAS* status. We are hesitant to call CGA a bona fide EGFR ligand at this time. It has low sequence similarity to EGF and high concentrations of CGA were required to induce EGFR phosphorylation. It lacks a transmembrane domain and the conserved spacing of the 3 disulfide bonds found in all other EGFR ligands. Nevertheless, our results suggest that a closer look at CGA as a potential EGFR ligand is warranted.

Targeting CGA may be a viable option for overcoming chemoresistance. Clinically, unbound CGA has been detected at low levels in urine and plasma from pregnant women as well as in placental explants and pituitary tissues (25). Elevated CGA levels were found in patients with trophoblastic malignancies (48). An early study reported that patients with trophoblastic tumors who experienced recurrences after chemotherapy showed higher blood CGA levels than those who remained in remission (49). This study implies a link between CGA and chemotherapy; however, the specific role and mechanism of CGA involvement in chemoresistance has not been previously elucidated. We used CRISPR-based gene editing, siRNA-mediated gene silencing, and anti-EGFR therapy to interfere with the transcription, translation, and function of CGA, respectively, all of which enhanced the effectiveness of chemotherapy. Nevertheless, a complete understanding of the biology of CGA is a prerequisite for its use in clinical practice. It is still unknown how unbound CGA is secreted from cells. Previous studies have revealed that additional carbohydrates placed on CGA interfere with its ability to bind to the β-subunit so that dimerization cannot take place (50). Even in the 2 MDR cell sublines, secreted CGA exhibited a slight difference in MW (Figure 1B), suggesting that the posttranslational modifications of CGA can be heterogeneous. Going forward, characterization of the different forms of CGA and their biological functions will aid in understanding the diverse roles of CGA in cancer.

The classical role of the GATA family is to mediate transcriptional regulation in hematopoiesis (GATA1–3) and cardiac development (GATA4–6) (51). As a hematopoietic factor, GATA2 participates in maintaining the proliferation and differentiation of hematopoietic cells, with its dysregulation contributing to leukemogenesis (52). More recently, an increase in GATA2 expression and transcriptional activity was found to confer drug resistance to leukemia and prostate cancer cells (53, 54). Our work suggests that GATA2 functions as a “regulator” in the CGA/EGFR/GATA2 circuit whereby GATA2 regulates CGA transcription, while GATA2 itself is under the control of CGA-mediated EGFR signaling, as well as an autoregulation mechanism during chemotherapy exposure. In our model, GATA2 expression and its accessibility to chromatin were induced by sublethal doses of chemotherapy. Based on this hypothesis, the question remains as to how cancer cells acquire chemoresistance in the setting of conventional chemotherapeutic dosing regimens. This may be due to the presence of genetically diverse clones within a tumor, with each population responding differently to chemotherapy, resulting in the emergence of high-GATA2-expressing clones. Another possibility is the complexity of the in vivo microenvironment, which impedes drug delivery into solid tumors and leads to concentration differences (55), suggesting that current treatment modalities still have potential for improvement.

## Methods

Further information can be found in Supplemental Methods.

### Secreted protein preparation.

Cells cultured with complete medium were washed with PBS, replaced with FBS-free medium, and cultured for another 24 hours, after which the medium was collected. After filtration with 0.45 μm filters to remove cell debris, the medium was concentrated at 4,000*g* for 1 hour by using 3 KD Ultra Centrifugal Filters (Millipore) to a desired volume.

### Cell culture and treatment.

SGC7901, BGC823, and MKN45 cells were obtained from the China Infrastructure of Cell Line Resources. NCI-N87, HEK293T, and HEK293FT cells were obtained from the American Type Culture Collection. All cells were maintained and passaged at the State Key Laboratory of Cancer Biology (CBSKL). The MDR SGC7901^ADR^ and SGC7901^VCR^ cells were previously established in the CBSKL from SGC7901 cells (23). All cell lines were authenticated by short tandem repeat analysis and were frequently checked for their morphological features and functionalities. All cell lines were confirmed to be free of mycoplasma contamination. Cells were grown in Dulbecco’s modified Eagle’s medium (DMEM) supplemented with 10% FBS, glutamine, nonessential amino acids, and antibiotics in a 5% CO_2_ incubator at 37°C. To maintain the MDR phenotype, 0.5 μg/mL Adriamycin or 1 μg/mL vincristine was added to the culture medium of SGC7901^ADR^ or SGC7901^VCR^ cells, respectively. Unless specified otherwise, cells were treated with rCGA (20 μg/mL) and EGF (50 ng/mL) after serum starvation for 12 hours. Drugs were used as follows: fluorouracil (10 μg/mL), Adriamycin (10 μg/mL), paclitaxel (10 μg/mL), cetuximab (10 μg/mL), erlotinib (20 nM), BFA (5 nM), BMA (1 μM), MG132 (10 μM), PD98059 (5 μM), SB202190 (10 μM), SP600125 (5 μM), and LY294002 (10 μM).

### Human tissue and plasma samples.

All human GC samples were obtained from the Xijing Hospital of Digestive Diseases. The pathological information for the samples was provided by the Department of Pathology. In total, we collected 37 pairs of tumor specimens before and after neoadjuvant chemotherapy. Prechemotherapy-treated specimens were obtained by biopsy under gastroscopy of patients with GC, and postchemotherapy specimens were collected at the time of surgery. Human plasma samples were provided by the Biobank of CBSKL. A total of 139 human plasma samples were obtained from 97 patients with GC who had received 4 to 12 cycles of chemotherapy and from 42 patients newly diagnosed with GC before receiving treatment. Response Evaluation Criteria in Solid Tumors (RECIST, version 1.1; ref. 56) was used to evaluate patients’ response to chemotherapy. Fifty-seven plasma samples from healthy donors were used as normal controls. No significant difference in age, sex, or TNM stage was observed between the normal controls and GC patients.

### Animal studies.

For the in vivo tumorigenesis assays, 6- to 8-week-old female athymic BALB/c nude mice were purchased from Beijing Vital River Laboratory Animal Technology. Mice were maintained under a 12-hour light/12-hour dark cycle and on a standard chow diet at a specific pathogen–free facility at the Experimental Animal Center of the Fourth Military Medical University. Suspensions of the cells used were subcutaneously injected into the posterior flanks of mice (5 × 10^6^ tumor cells/150 μL PBS per spot; 5–8 mice in each group). When the tumors reached a predetermined size (approximately 100 mm^3^), the mice were randomized into control and treatment groups. For the generation of PDX models, 6- to 8-week-old male NOD-*Prkdc*^em1IDMO^-*Il2rg*^em2IDMO^ (Hu-CD34 NPI) mice were purchased from Beijing IDMO. Fresh GC tissues were collected under the approval of the Medical Ethics Committee of Xijing Hospital and informed consent was obtained from all patients. Gastric tumor tissues were collected at the time of surgery and the general information of patients is listed in Supplemental Table 3. Tumor fragments (approximately 3 × 3 × 3 mm^3^) were transplanted into NPI mice (*n =* 3–5 in each case). A part of the tumor was cryopreserved to establish a live-tumor bank, and the rest was reimplanted. P0 denotes the original patient-derived tumor tissue, with subsequent passages numbered consecutively. Early generations (P2 to P4) of the PDX models were treated with saline or chemotherapeutic agent when the tumors reached the predetermined size (approximately 80–200 mm^3^), and the tumor size was monitored as an indicator of drug response using a bilateral caliper. Tumor volume was calculated using the following formula: tumor maximum diameter (*L*) × the right-angle diameter to that axis (*W*)^2^/2. After 3 to 5 weeks of treatment, mice were sacrificed according to institutional ethical guidelines. Postmortem examination included tumor size and tumor weight measurements, and then tumors, livers, and kidneys were paraffin embedded for further investigation.

### MS.

For secretome analysis, secreted proteins from SGC7901, SGC7901^ADR^, and SGC7901^VCR^ cells were collected using 3 KD Ultra Centrifugal Filters (Millipore) and the concentration was determined by using the BCA assay. The protein samples were prepared following a previous report with some modifications (57, 58). A detailed description is included in the Supplemental Methods.

### Protein extraction and immunoblotting.

Proteins were extracted from cultured cells or concentrated FBS-free media using RIPA buffer (150 mM NaCl, 50 mM Tris, 1% Nonidet P-40, pH 7.5) supplemented with protease and phosphatase inhibitors (Roche) and boiled with SDS loading buffer for 10 minutes. The denatured proteins were resolved in a 4% to 20% SDS-PAGE gel for immunoblotting analysis with the indicated antibodies listed in Supplemental Table 10. The blotted bands were visualized on X-ray films or by the Bio-Rad ChemiDoc XRS+ Imaging System. The Human Phospho-RTK Array Kit was purchased from R&D Systems.

### RNA extraction and RT-qPCR.

Total RNA was extracted from cells using TRIzol reagent (Invitrogen). cDNA synthesis was carried out using the PrimeScript RT Reagent Kit (TaKaRa) and qPCR was performed using the SYBR Premix Ex Taq Kit (TaKaRa). Fluorescence was measured and C_T_ values were calculated using a LightCycler 480 system (Roche). The PCR primers used are listed in Supplemental Table 11. Primers for the miRNAs were purchased from Guangzhou RiboBio.

### IF.

Cells were seeded on a Lab-Tek chamber slide (Nunc) and fixed in 4% paraformaldehyde for 20 minutes. The cells were permeabilized using 0.1% Triton X-100 in PBS for 15 minutes and blocked with 2% BSA in PBS for 30 minutes. Incubation of primary antibodies was performed at 4°C overnight, and secondary antibodies were incubated at room temperature for 1 hour. The nuclei were stained with DAPI and fluorescence images were taken on the Nikon A1 Confocal Laser Microscope System.

### IHC.

Paraffin-embedded specimens were serially sectioned, deparaffinized, and treated with 3% H_2_O_2_ to block endogenous peroxidase activity. Slides were immersed in an antigen retrieval buffer (10 mM sodium citrate acid, 0.05% Tween 20, pH 6.0) and heated to 120°C for 20 minutes and then allowed to cool to room temperature. Incubation of primary antibodies was performed at 4°C overnight, and secondary antibodies were incubated at room temperature for 1 hour. Quantification of protein expression was based on the intensity and extent of staining according to the histological scoring method as previously described (59). Briefly, the intensity of staining was determined as follows: 0, no staining; 1, weak staining; 2, moderate staining; and 3, strong staining. The mean proportion of stained cells per specimen was determined semiquantitatively. The IHC score for each specimen was calculated as the product of staining intensity times the percentage of positive tumor cells. Tissue slides and tissue microarrays were scanned using the Olympus VS120 virtual slide scanning system.

### Constructs, oligonucleotides, and cell transfection.

Expression vectors encoding CGA and CGA plus its 3′-UTR were constructed by subcloning the CGA ORF with or without its 3′-UTR into a modified pHL-sec vector containing a C-terminal 6×His tag. An expression vector encoding the CGA-GST fusion protein was constructed by subcloning the CGA ORF into a pcDNA3.1 vector containing a C-terminal GST tag. For the ΔSP, N52Q, N78Q, and DM CGA constructs, mutations were generated by QuickChange site-directed mutagenesis. To establish CGA stably transfected cells, CGA cDNA was subcloned into a pLVX-IRES-Puro vector. CGA stably expressing cells were established by lentivirus infection and selected with puromycin. An expression vector encoding GATA2 was constructed by subcloning the GATA2 cDNA into a pcDNA3.1 vector. Expression vectors encoding the FL, ECD (residues 1–668), or intracellular domain (ICD) (residues 646–1210) of EGFR were constructed by subcloning the corresponding EGFR cDNA fragments with a C-terminal Flag tag into a pcDNA3.1 vector. Synthetic mimics of miR-17-3p, miR-630, miR-708-3p, miR-761, and a negative control were purchased from Guangzhou RiboBio. siRNAs against CGA and GATA2 and a scrambled control were purchased from GenePharma. All the plasmids and oligonucleotides were transfected into the target cells using the JetPRIME (Polyplus Transfection) reagent following the manufacturer’s instructions.

### Generation of CRISPR/Cas9 knockout cell lines.

Three guide RNAs (gRNAs) targeting *CGA* were designed using the CRISPRdirect web server (https://crispr.dbcls.jp), and the target sequences in *CGA* are 5′-TTGCCCAGAATGCACGCTAC-3′, 5′-GAGCATATCCCACTCCACTA-3′, and 5′-CCATTCCGCTCCTGATGTGC-3′. The human codon–optimized Cas9 (hCas9) and GFP-targeting gRNA-expressing plasmids (gRNA_GFP-T1) were purchased from Addgene. The GFP-targeting sequence in the gRNA vector was replaced by QuickChange site-directed mutagenesis. To construct the knockout cell lines, the gRNA-expressing plasmid, hCas9 plasmid, and pEGFP-C1 vector were cotransfected into SGC7901^ADR^ and SGC7901^VCR^ cells. GFP-positive cells were sorted into single clones into a 96-well plate by flow cytometry. Single clones were screened by the T7 endonuclease I–cutting assay or anti-CGA immunoblotting. The knockout clones were confirmed by DNA sequencing.

### Cell viability measurement.

Cells were seeded into a 96-well plate and allowed to adhere to the wells. After the indicated treatment, cells were washed with PBS, and cell viability was measured using a Cell Counting Kit-8 Assay (Dojindo). Briefly, a mixture of cell counting solution and DMEM at a ratio of 1:10 was dispensed into each well, and samples were then incubated at 37°C for 2 hours. The absorbance values were read at 450 nm using a Thermo Fisher Scientific Varioskan Flash multimode reader.

### Apoptosis analysis.

After the indicated treatment, cells were harvested, resuspended in staining buffer, and examined using an Annexin V–FITC Early Apoptosis Detection Kit (Cell Signaling Technology). Then, the cells were sorted by flow cytometry, and the data were analyzed using EXPO32 ADC software (Beckman Coulter). Cells staining positive for Annexin V–FITC and negative for propidium iodide were considered to have undergone apoptosis.

### Purification of recombinant protein.

rCGA was expressed and purified from HEK293FT cells as previously described (60). Briefly, CGA containing a C-terminal 6×His tag secreted in the culture medium was captured onto a nickel affinity column. The eluted CGA protein containing an engineered enterokinase site at the C-terminus was digested with enterokinase to remove the 6×His tag. Subsequently, cation-exchange chromatography (GE Healthcare Life Sciences) was carried out to further purify the CGA protein. The prokaryotic CGA protein purified from *E*. *coli* was purchased from Sino Biological.

### Immunoprecipitation.

To detect the interaction between CGA and EGFR, 15-cm dishes seeded with SGC7901 cells expressing Flag-tagged EGFR fragments were incubated with His-tagged CGA for 30 minutes. Cells were lysed in RIPA buffer containing protease inhibitor cocktail. The anti-EGFR antibody was mixed with Protein G Sepharose (Sigma-Aldrich) and incubated at 4°C for 1 hour. Antibody-Sepharose conjugates, anti-Flag M2 Affinity Agarose Gel (Sigma-Aldrich), or His-Tag Dynabeads (Invitrogen) were added to the soluble fraction of centrifuged cell lysates and incubated at 4°C for 2 hours. The immunoprecipitants were washed 5 times with RIPA buffer, followed by immunoblotting analysis.

### Molecular docking.

The 3D structures of the ECD of EGFR (PDB ID: 4UV7) and CGA (PDB ID: 1E9J) were downloaded from RCSB Protein Data Bank (https://www.rcsb.org/). Protein-protein docking between the ECD of EGFR and CGA was simulated online by ClusPro server4-8 (https://cluspro.org) with the ECD of EGFR selected as the receptor and CGA as the ligand. Molecular graphics were generated using PyMOL as previously described (61).

### SPR analysis.

Analyses of the binding interaction between CGA and EGFR as well as the binding kinetics were performed using a BIAcore S200 SPR instrument (GE Healthcare) at room temperature. The recombinant ECD of EGFR was immobilized on a CM5 sensor chip using amine coupling. Purified rCGA was injected at indicated concentrations at a flow rate of 30 μL/min for 60 seconds. The dissociation was monitored for 100 seconds. The resulting data after subtracting control values were analyzed by fitting to a 1:1 Langmuir binding model using BIAcore S200 evaluation software.

### GST pull-down assay.

WT or mutant CGA-GST fusion protein, or GST alone, was expressed in HEK293FT cells and affinity-purified using glutathione magnetic agarose beads (Millipore). For an in vitro GST pull-down assay, an expression vector encoding a Flag-tagged ECD of EGFR was transfected into HEK293T cells. The whole-cell lysates were extracted using NP-40 lysis buffer and were incubated with affinity-purified fusion proteins bound to glutathione magnetic agarose beads. The bound proteins were resolved by electrophoresis and examined by immunoblotting.

### RNA sequencing and data analysis.

A detailed description is included in the Supplemental Methods. RNA sequencing data are available at the NCBI’s GEO database with accession GSE193739.

### Luciferase reporter assay.

For the luciferase reporter assay measuring promoter activities, the WT, truncated, and mutant constructs of *CGA* promoter fragments were subcloned upstream of the firefly luciferase reporter in a pGL3-Basic vector. Cells were cotransfected with pGL3-CGA promoter fragments, pRL-SV40 *Renilla* luciferase reporter, and pcDNA3.1-GATA2 plasmid or empty vector control. For 3′-UTR luciferase reporter assays, the WT and mutant constructs of the 3′-UTR of CGA were subcloned into a psiCHECK-2 vector. Cells were cotransfected with psiCHECK-2-CGA 3′-UTR WT or mutant plasmids and miRNA mimics or a negative control. The firefly and *Renilla* luciferase activities were measured using the Dual-Luciferase Reporter Assay System (Promega). Firefly luciferase activity was normalized to *Renilla* activity and is presented as relative luciferase activity.

### ChIP.

ChIP was performed using a Pierce Agarose ChIP Kit following the manufacturer’s instructions. Briefly, cells were cross-linked with 1% formaldehyde for 10 minutes at room temperature and quenched in glycine. The anti-GATA2 antibody was used for immunoprecipitation, and a nonspecific antibody against IgG served as the negative control. Ten percent of the chromatin sample prior to immunoprecipitation was used as an input control. The precipitated DNA was recovered and subjected to qPCR to amplify the binding sites within the *CGA* promoter region or around the genomic *GATA2* locus with the primers listed in Supplemental Table 11. A primer pair –5.6 kb from the *CGA* promoter was used as a control.

### EMSA.

Nuclear protein was extracted using the NE-PER Nuclear and Cytoplasmic Extraction Reagents Kit (Thermo Fisher Scientific). A LightShift Chemiluminescent EMSA Kit (Thermo Fisher Scientific) was used according to the manufacturer’s protocol. Nuclear extracts were incubated with a biotin-labeled probe for GATA-binding elements, poly(dI-dC), and the binding buffer for 30 minutes at room temperature. For the binding competition experiment, an excess (200-fold) of unlabeled cold competitor probe or mutant probe was added into the reaction mixture. Bound DNA complexes were resolved in polyacrylamide gels and transferred to a nylon membrane. Nylon membranes were cross-linked, and chemiluminescent detection was performed. The probe sequences used are listed in Supplemental Table 11.

### ELISA.

A human CGA ELISA Kit (Novus) was used to quantify human plasma CGA levels. Briefly, plasma samples in triplicate were added to 96-well plates, and then the detection solutions and wash buffers were dispensed into each well in accordance with the manufacturer’s instructions. The absorbance values of each well were read at 450 nm using a Thermo Fisher Scientific Varioskan Flash multimode reader.

### Production of miRNA prodrugs.

The expression and purification of the recombinant Sephadex aptamer-tagged methionyl-tRNA (MSA)/miR-708-3p, MSA/miR-761, and control tRNA/MSA were conducted as previously described (62–64). A detailed description is included in the Supplemental Methods.

### Statistics.

Statistical analysis was performed using SPSS 18.0 (IBM) and R (version 3.3.1; https://cran.r-project.org/). A *P* value of less than 0.05 was considered statistically significant. Data are presented as mean ± SEM. Two-tailed Student’s *t* test, ANOVA (Dunnett’s or Bonferroni’s post hoc test), nonparametric signed-rank test, χ^2^ test, and Pearson’s correlation coefficient were used according to the type of experiment and are indicated in the figure legends.

### Study approval.

The study was approved by the Medical Ethics Committee of Xijing Hospital, with written informed consent obtained from all patients. All animal experiments were conducted under protocols approved by the Institutional Animal Care and Use Committee at the Fourth Military Medical University (Xi’an, China).

## Author contributions

XZ, YN, DF, and RJC designed the research. TC, XZ, YL, QW, HQ, HL, SEG, BS, WZ, JD, and FD performed experiments, analyzed data, and prepared figures and tables. HG, MG, AQ, and YT contributed to analytical tools and/or new reagents. XZ, YL, XW, CL, KW, DF, YN, and RJC analyzed the data and provided critical input. XZ and RJC wrote the manuscript. XZ conceived the project, supervised, and coordinated all aspects of the work.

## Supplementary Material

Supplemental data

## Version 1 03/15/2022

Electronic publication
