# Supplementary material for: A CGA/EGFR/GATA2 positive feedback circuit confers chemoresistance in gastric cancer
Source: J Clin Invest. 2022 Mar 15;132(6):e154074. doi: 10.1172/JCI154074 (PMC8920335; doi:10.1172/JCI154074)
Supplement: Supplemental data [file jci-132-154074-s139.pdf]

Supplemental Material

A glycoprotein hormones alpha subunit/EGFR/GATA2 positive feedback circuit confers chemoresistance in gastric cancer

Tianyu Cao, Yuanyuan Lu, Qi Wang, Hongqiang Qin, Hongwei Li, Hao Guo, Minghui Ge, Sarah E. Glass, Bhuminder Singh, Wenyao Zhang, Jiaqiang Dong, Feng Du, Airong Qian, Ye Tian, Xin Wang, Cunxi Li, Kaichun Wu, Daiming Fan, Yongzhan Nie, Robert J. Coffey and Xiaodi Zhao

Supplementary Methods

Mass spectrometry

The proteins were reduced by dithiothreitol at 37°C for 2 h and alkylated by iodoacetamide in the dark at RT for 30 min. Then, trypsin was added at a ratio of 1:50 (trypsin/proteins, w/w) and incubated at 37°C overnight. Trypsin was added again at the same weight ratio and incubated for another 4 h. The tryptic digests were desalted by an SPE column (Waters) and dried for further MS analysis. For glycosylation site identification, the CGA glycopeptide enrichment was performed with click maltose-hydrophilic interaction liquid chromatography (HILIC) materials. The prepared materials (5.0 mg) were washed with 80% acetonitrile (ACN) with 1% trifluoroacetic acid, and the digestions of CGA digests were mixed with HILIC materials. After incubation at RT for 40 min, the mixtures were translated into a tip and the supernatant was removed after centrifugation at 4000× g for 10 min. The HILIC tip was further washed three times with 200 µl of loading buffer. Finally, the enriched N-linked glycopeptides were eluted with 100 µl of 30% ACN with 0.1% formic acid (FA). The eluted samples were dried and treated with PNGase F (500 units) in 10 mM NH_4HCO_3 (pH 7.8) at 37°C overnight. The deglycosylated peptides were dried and used for the identification of the glycosylation sites by further MS analysis.

The LC-MS/MS analyses were performed on a Q-Exactive HF mass spectrometer equipped with an Ultimate 3000 system (Thermo) for separation. The LC-MS/MS system contained a C18 capillary trap column (200 µm i.d., C18 AQ beads (1.9 µm, 120 Å)) and a 15 cm C18 capillary analysis column (150 µm i.d., C18 AQ beads (1.9 µm, 120 Å)). Mobile

phases A (98% H_2O, 2% acetonitrile, 0.1% FA) and B (80% acetonitrile, 20% H_2O, 0.1% FA) were used to develop a gradient. For the deglycosylated peptides, the reversed-phase gradient was developed at 600 nl/min as follows: loading sample for 12 min, 4 to 45% buffer B for 25 min, 45 to 90% buffer B for 3 min, 90 to 90% buffer B for 10 min, 90 to 4% buffer B for 0.5 min and 4 to 4% buffer B for 9.5 min. The temperature of the ion transfer capillary was 250°C, and the normalized collision energy was set to 27%. The mass resolution was set to 70,000 for full MS and 15,000 for higher-energy collisional dissociation MS/MS. Survey full scan MS was acquired from m/z 400 to 2,000, and the 15 most intense ions were selected for the MS/MS scan. The dynamic exclusion was set as follows: repeat count, 1; duration, 22 s; exclusion list size, 500; and exclusion duration, 30 s. For glycosylation site analysis, the mass spectrometric data of deglycosylated peptides acquired by MS was directly searched by using pFind (version 3.1.5). The following parameters were used for the search: mass tolerances were 20 ppm for both the precursor and fragments; for trypsin digested samples, enzyme specificity was set to KR/P with up to 2 missed sites; cysteine residues were set as fixed modifications (C, +57.022 Da), and the N-linked glycosylation sites were obtained with sequon (N-X-S/T, X≠P).

RNA sequencing and data analysis

Total RNA from cells was extracted with TRIzol and assessed with an Agilent 2100 BioAnalyzer (Agilent Technologies) and Qubit Fluorometer (Invitrogen). All RNA samples that passed quality tests with an RNA integrity number (RIN) > 7.0 and a 28S:18S

ratio > 1.8 were used for RNA sequencing. Sequence libraries were generated and sequenced by CapitalBio Technology. RNA sequencing reads were aligned to the human genome hg38 using HISAT2 with default parameters, and the number of reads mapped to each gene was calculated by HISAT (http://www.ccb.jhu.edu/software/hisat/). Differentially expressed genes (DEGs) between samples were detected by edgeR based on a negative binomial distribution. The p values were adjusted by Benjamini and Hochberg's multiple test correction procedures. By searching the ENSEMBL, NCBI, UniProt, GO and KEGG databases, a Basic Local Alignment Search Tool (BLAST) alignment was performed to determine the functional annotation of DEGs, and the best matches were selected. KEGG pathway enrichment analysis was performed for the DGEs using KOBAS 3.0 software.

Production of miRNA prodrugs

MSA/miR-708-3p-, MSA/miR-761- and tRNA/MSA-expressing plasmids were transformed into HST08 *E. coli* competent cells. Separation of target RNAs from total bacterial RNA was achieved on an Enrich-Q 10 × 100 column by using the NGC QUEST 10 PLUS fast protein liquid chromatography (FPLC) system (Bio-Rad). The system was first equilibrated with Buffer A (10 mM sodium phosphate, pH 7.0) at a constant flow rate of 2.5 ml/min for 4.4 min and then subjected to the following gradient elution: 64% Buffer B (Buffer A + 1 M sodium chloride, pH 7.0) for 10 min, 64-78% Buffer B for 8 min, and then 100% Buffer B for 3 min. FPLC traces were monitored at 260/280 nm using a UV/Vis

detector. After the confirmation of target RNA by urea-PAGE analyses, fractions were pooled, precipitated by ethanol, desalted and concentrated with centrifugal filters. RNA purities were verified by a high-performance liquid chromatography assay. The purified MSA/miR-708-3p, MSA/miR-761 and tRNA/MSA were formulated with the in vivo-jetPEI (Polyplus Transfection) reagent and administered intratumorally into mice.

Figure S1

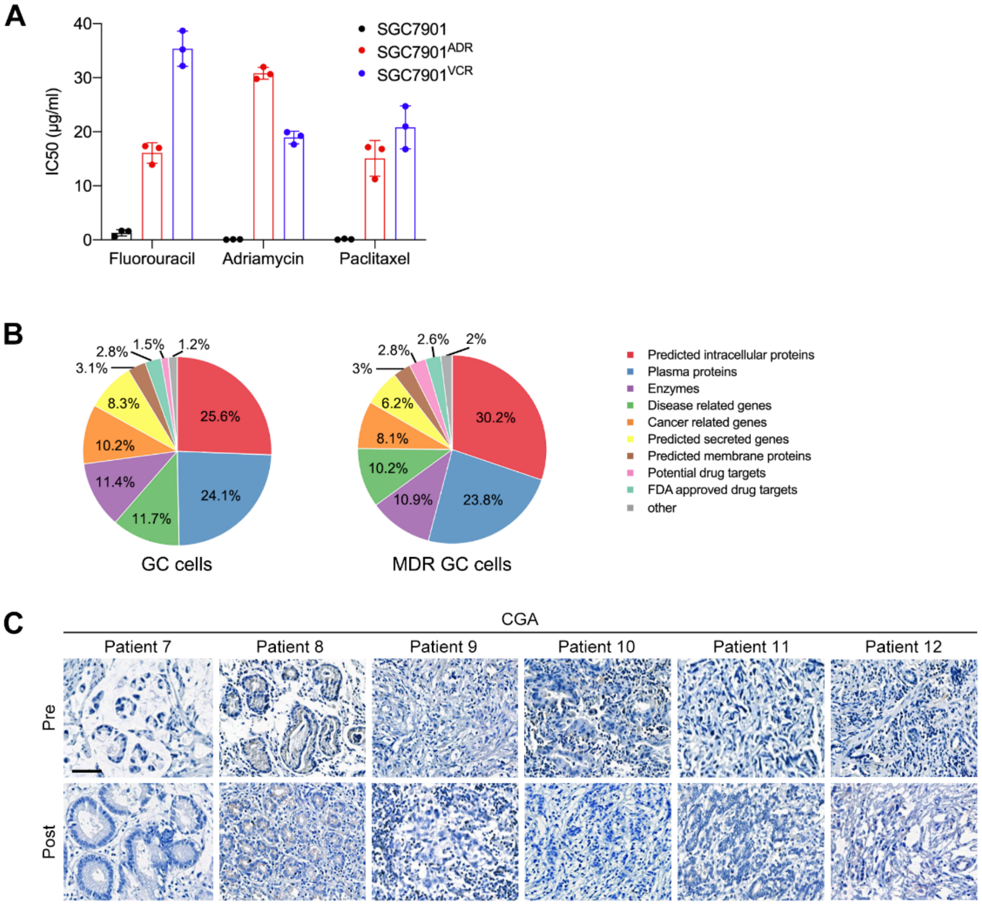

Fig. S1. CGA is upregulated in chemoresistant gastric cancer (GC) cells and tissues, related to Fig. 1.

(A) Half maximal inhibitory concentration (IC50) values for indicated chemotherapeutic agents were measured in chemosensitive SGC7901 cells and multi-drug resistant (MDR) $SGC7901^{ADR}$ and $SGC7901^{VCR}$ cells. Data are presented as mean ± SEM. **(B)** Functional analysis of the secreted proteins from GC cells (SGC7901) and MDR GC cells ($SGC7901^{ADR}$ and $SGC7901^{VCR}$) annotated as blood proteins. The color code represents ten functional classes. **(C)** IHC staining of CGA in all the six human GC specimens obtained from patients who responded to chemotherapy (scale bar, 50 μm).

Figure S2

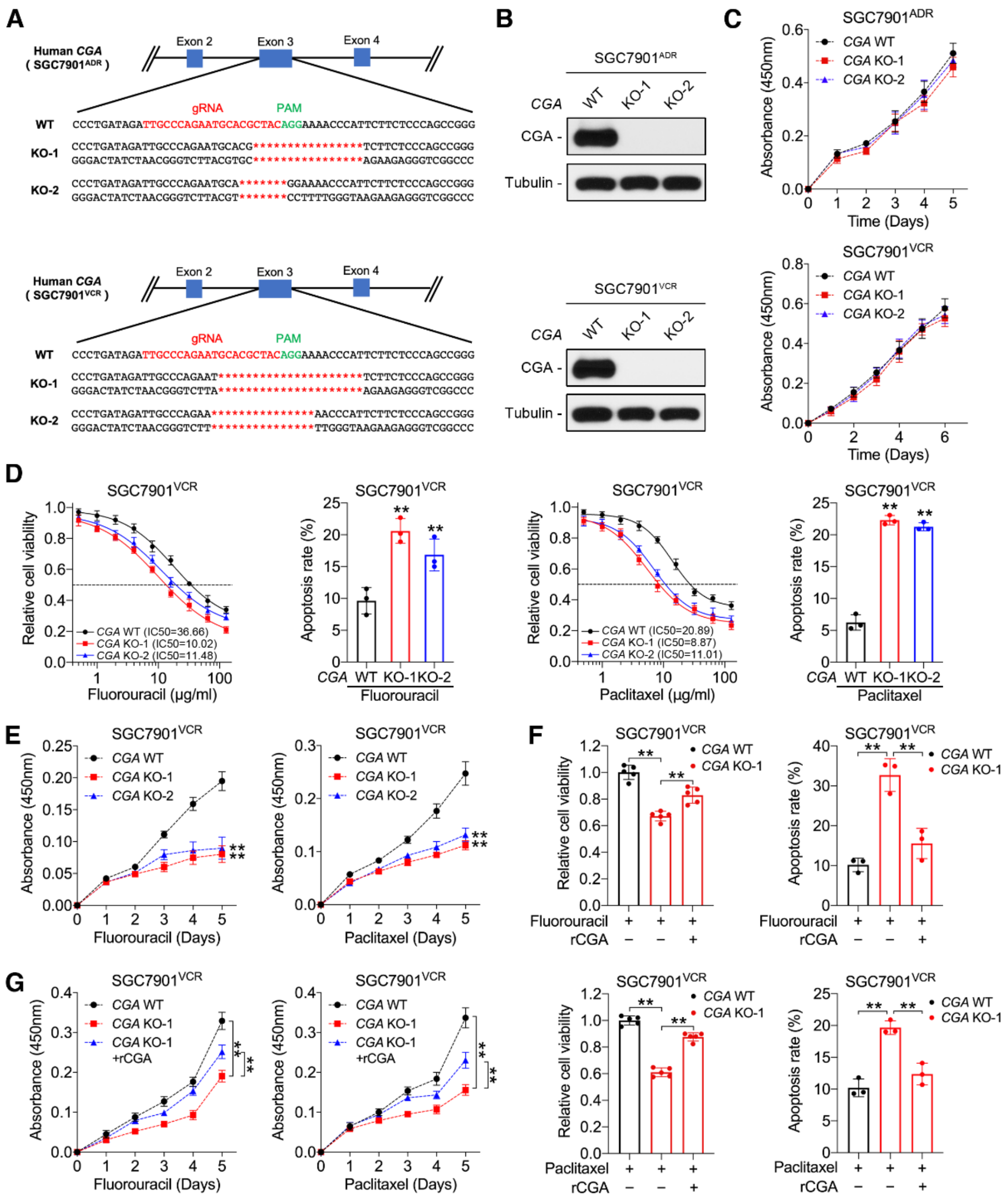

Fig. S2. CGA is important to maintain chemoresistance in GC cells, related to Fig. 2.

(A) *CGA*$^{-/-}$ MDR cells were generated by CRISPR/Cas9-mediated genome editing. Sequence of the targeted region and the two knockout alleles (KO-1 and KO-2) are shown. **(B)** Immunoblotting of CGA expression in *CGA* WT and KO MDR cells. **(C)** Growth

curves of *CGA* WT and KO MDR cells in the absence of chemotherapy. **(D)** *CGA* WT or KO SGC7901^{VCR} cells were treated with fluorouracil (10 μg/ml) or paclitaxel (10 μg/ml). The IC50 values and apoptosis were measured. **(E)** Growth curves of *CGA* WT and KO SGC7901^{VCR} cells in the presence of fluorouracil or paclitaxel. **(F, G)** Viability, apoptosis (F) and growth curves (G) of *CGA* WT or KO SGC7901^{VCR} cells that were treated with or without recombinant human CGA (rCGA, 20 μg/ml) in the presence of fluorouracil or paclitaxel. Data are presented as mean ± SEM. **p < 0.01 by one-way ANOVA followed by Dunnett's multiple comparison in (D-G).

Figure S3

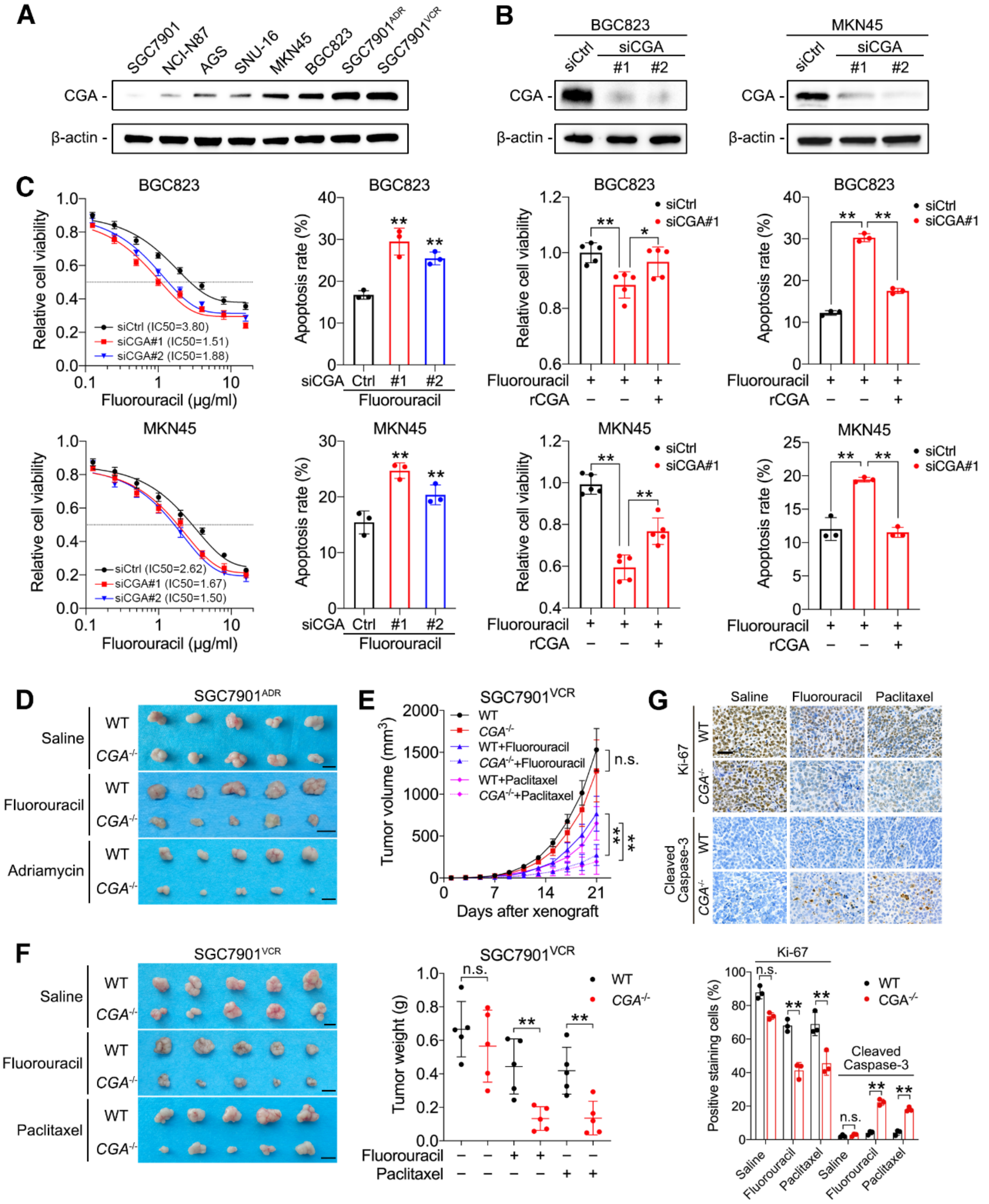

Fig. S3. CGA is important to maintain chemoresistance in GC cells both *in vitro* and *in vivo*, related to Fig. 2.

(A) Immunoblotting of CGA in a panel of GC cell lines. **(B)** Immunoblotting of CGA in

BGC823 and MKN45 cells transfected with two independent siRNAs against CGA (siCGA) or a control siRNA (siCtrl). **(C)** BGC823 and MKN45 cells transfected with siCGA or siCtrl were treated with fluorouracil. The IC50 values and apoptosis were measured with or without rCGA treatment. **(D)** Xenograft tumors of *CGA* WT and KO SGC7901^{ADR} cells isolated on day 21 after the indicated treatment. **(E-G)** *CGA* WT or KO SGC7901^{VCR} cells were injected subcutaneously into nude mice (n=5). After tumors were palpable, mice received indicated treatment every 3 days (fluorouracil, 20 mg/kg, i.p. injection; paclitaxel, 3 mg/kg, i.p. injection) and tumor size was monitored (E). Tumors were isolated on day 21 after treatment and tumor weight was measured (F). IHC staining of Ki-67 and cleaved Caspase-3 in tumors harvested from each group is shown and the percentages of Ki-67- and cleaved Caspase-3-positive cells were measured (G; scale bar, 50 μm). Data are presented as mean ± SEM. *p < 0.05, **p < 0.01 by one-way ANOVA followed by Dunnett's multiple comparison in (C), by repeated-measures ANOVA test followed by Dunnett's multiple comparison in (E) or by Student's *t* test in (F and G).

Figure S4

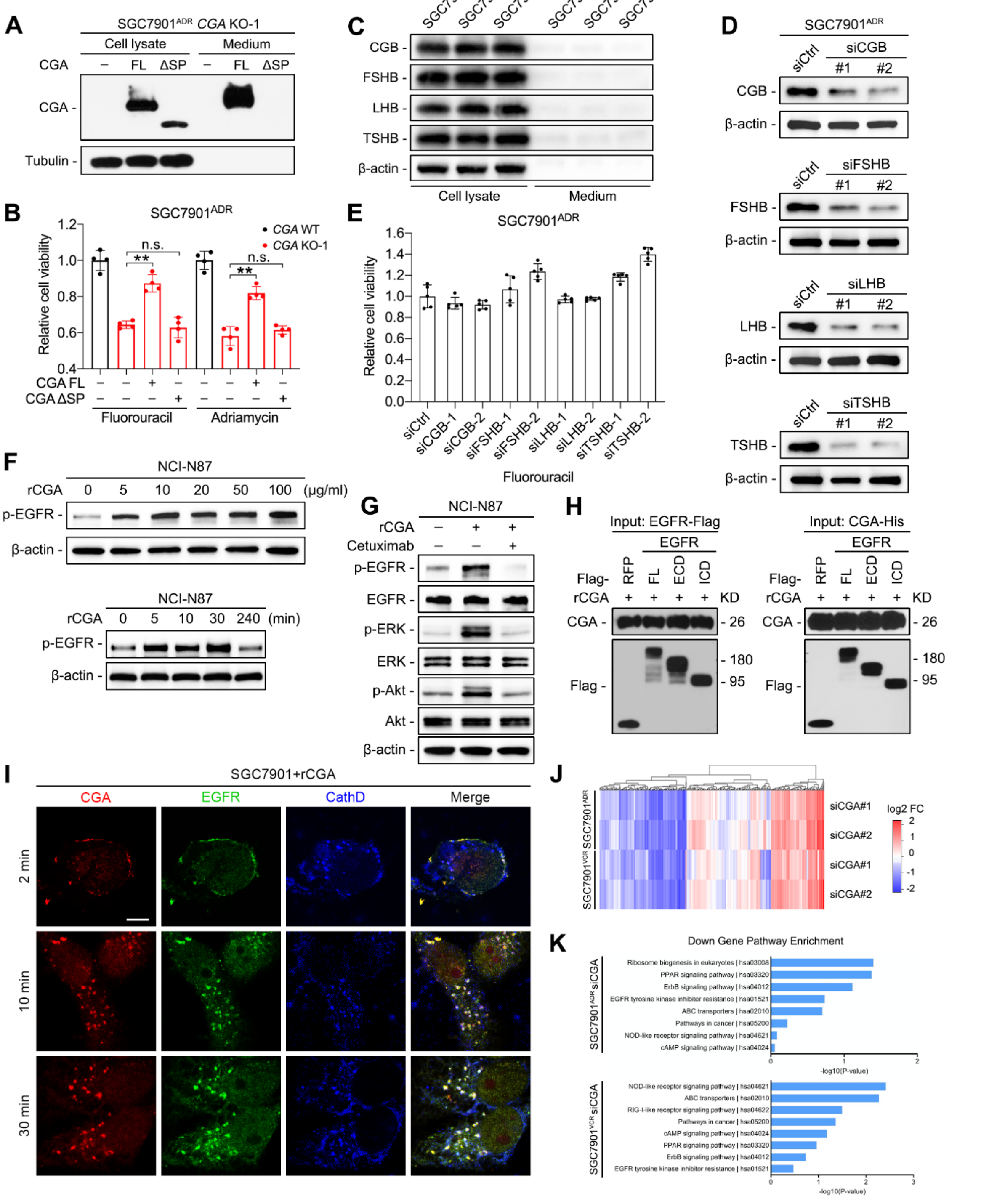

Fig. S4. CGA binds to EGFR and activates EGFR downstream signaling in GC cells, related to Fig. 3.

(A, B) Immunoblotting of CGA in *CGA*$^{-/-}$ SGC7901^{ADR} cells complemented with FL or ΔSP CGA (A), and viability of indicated cells treated with fluorouracil or adriamycin (B). **(C)** Immunoblotting of the β-subunits of hCG, FSH, LH and TSH in cell lysates and conditioned media from SGC7901 and MDR cells. **(D)** Immunoblotting of the β-subunits of hCG, FSH, LH and TSH from SGC7901^{ADR} cells transfected with their corresponding siRNAs or siCtrl. **(E)** Viability of SGC7901^{ADR} cells transfected with siRNAs against the β-subunits of hCG, FSH, LH and TSH. **(F)** Immunoblotting of p-EGFR in serum-starved NCI-N87 cells when treated with the indicated concentrations of rCGA (top) or treated with rCGA (20 μg/ml) at different time points (bottom). **(G)** NCI-N87 cells pretreated with cetuximab (10 μg/ml) followed by rCGA treatment and immunoblotting with indicated antibodies. **(H)** Total cell lysates were blotted as a control for immunoblotting analysis in Figure 3H. **(I)** Immunofluorescence (IF) staining of CGA, EGFR and lysosome marker cathepsin D (CathD) in SGC7901 cells after treatment with rCGA at 37°C for a time course (scale bar, 5 μm). **(J)** Heatmap of differentially expressed genes (fold change > 2 and $p < 0.01$) in MDR cells after CGA knockdown. **(K)** KEGG pathway enrichment analysis identified top de-enriched pathways following CGA knockdown. Data are presented as mean ± SEM. **$p < 0.01$ by one-way ANOVA followed by Dunnett's multiple comparison in (B). n.s., not significant.

Figure S5

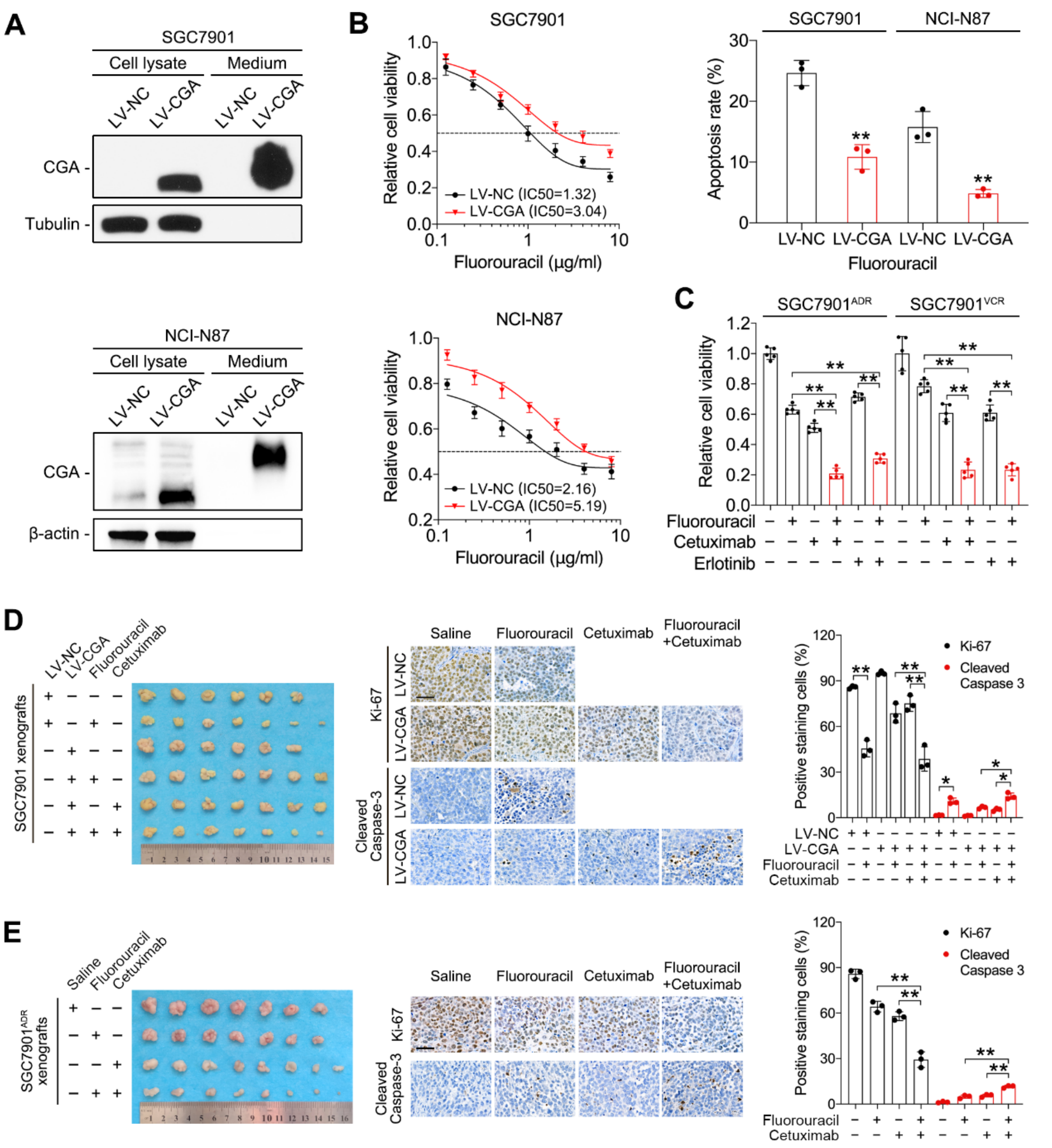

Fig. S5. CGA binds to EGFR and activates EGFR downstream signaling in GC cells, related to Fig. 3.

(A) Immunoblotting of CGA in cell lysates and conditioned media from SGC7901 and NCI-N87 cells infected with lentivirus expressing CGA (LV-CGA, MOI=10) or control lentivirus (LV-NC, MOI=10). **(B)** SGC7901 and NCI-N87 cells infected with LV-CGA or

LV-NC were treated with fluorouracil. The IC50 values and apoptosis were measured. **(C)** Viability of SGC7901^{ADR} and SGC7901^{VCR} cells treated with fluorouracil, cetuximab (10 μg/ml), erlotinib (20 nM) or a combination of them. **(D, E)** Xenograft tumors on day 24 for Figure 3K (D) and day 27 for Figure 3L (E). IHC staining of Ki-67 and cleaved Caspase-3 in tumors harvested from each group is shown and the percentages of Ki-67- and cleaved Caspase-3-positive cells were measured (scale bar, 50 μm). Data are presented as mean ± SEM. $^*p < 0.05$, $^{**}p < 0.01$ by Student's *t* test in (B, D and E) or by one-way ANOVA with Bonferroni's post hoc test in (C).

Figure S6

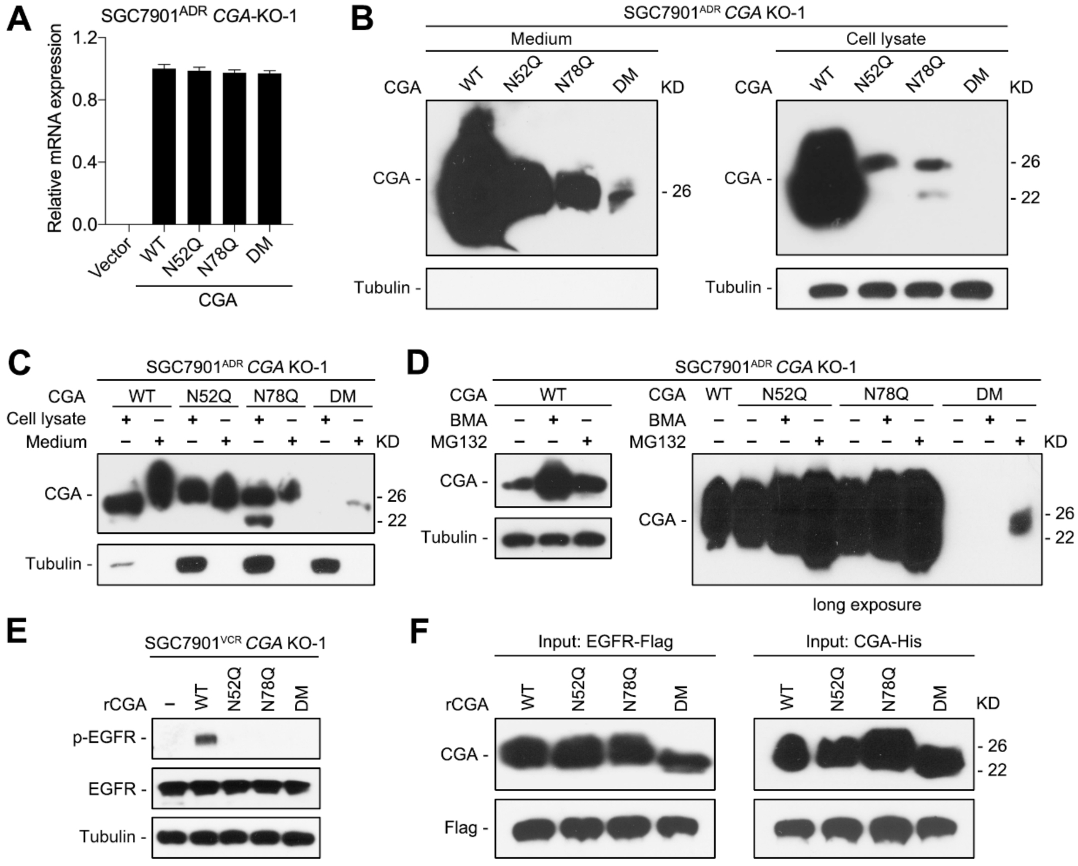

Fig. S6. N-linked glycosylation is required for CGA-induced chemoresistance, related to Fig. 4.

(A) RT-qPCR analysis of CGA expression in *CGA*$^{-/-}$ SGC7901^{ADR} cells transfected with WT, N52Q, N78Q or DM CGA. Data are presented as mean ± SEM. **(B, C)** Immunoblotting of CGA in cell lysates and conditioned media from *CGA*$^{-/-}$ SGC7901^{ADR} cells transfected with WT, N52Q, N78Q or DM CGA. **(D)** Immunoblotting of CGA in *CGA*$^{-/-}$ SGC7901^{ADR} cells transfected with WT, N52Q, N78Q or DM CGA followed by treatment with bafilomycin A1 (BMA, 1 μM) or MG132 (10 μM). **(E)** Immunoblotting of p-EGFR and EGFR in *CG*A$^{-/-}$ SGC7901^{VCR} cells treated with purified WT, N52Q, N78Q

or DM rCGA. **(F)** Total cell lysates were blotted as a control for immunoblotting analysis in Figure 4J.

Figure S7

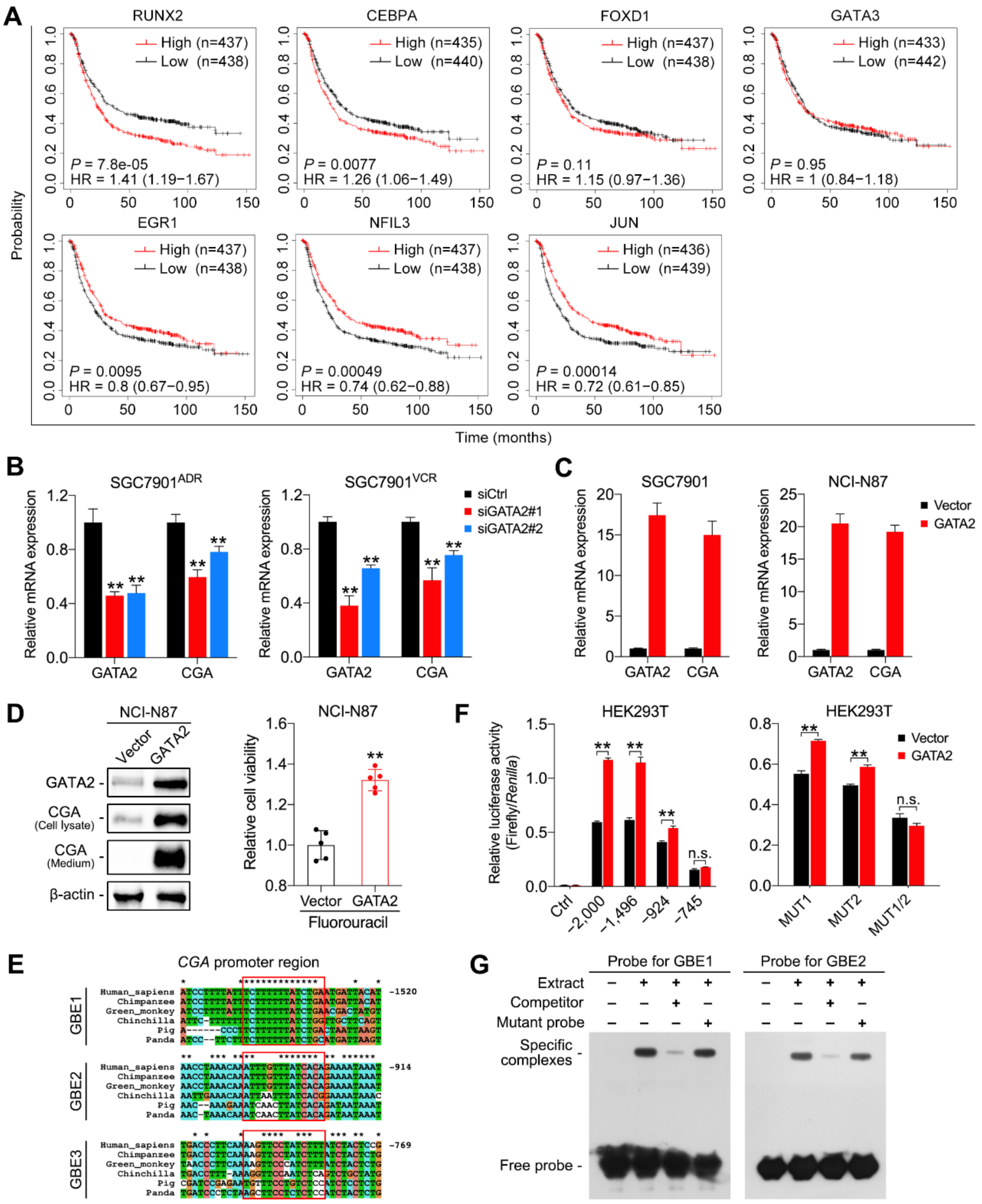

Fig. S7. The reciprocal positive regulation between GATA2 and CGA-EGFR signaling, related to Fig. 5.

(A) Kaplan-Meier analyses of correlations between expression levels of seven transcription

factor candidates and overall survival of GC patients in the KM plotter database. **(B, C)** RT-qPCR analyses of GATA2 and CGA expression in MDR cells transfected with siGATA2 or siCtrl (B) and in SGC7901 and NCI-N87 cells transfected with either a GATA2 expression vector or an empty vector (C). **(D)** Immunoblotting of GATA2 and CGA expression (left) and viability (right) in NCI-N87 cells transfected with either a GATA2 expression vector or an empty vector. **(E)** Multiple alignment of GATA2-binding elements (GBEs) homologues from different species. The GBEs are boxed in red, and the conserved flanking sequences are in orange, cyan and green. **(F)** The luciferase reporter driven by either the WT, deletion or mutant (MUT) promoter of CGA was transfected into HEK293T cells, and luciferase activity was measured with or without GATA2 co-transfection. **(G)** Electrophoretic mobility shift assay using nuclear extracts from SGC7901 cells and the indicated probes. Data are presented as mean ± SEM. $^{**}p < 0.01$ by one-way ANOVA followed by Dunnett's multiple comparison in (B) or by Student's *t* test in (D and F).

Figure S8

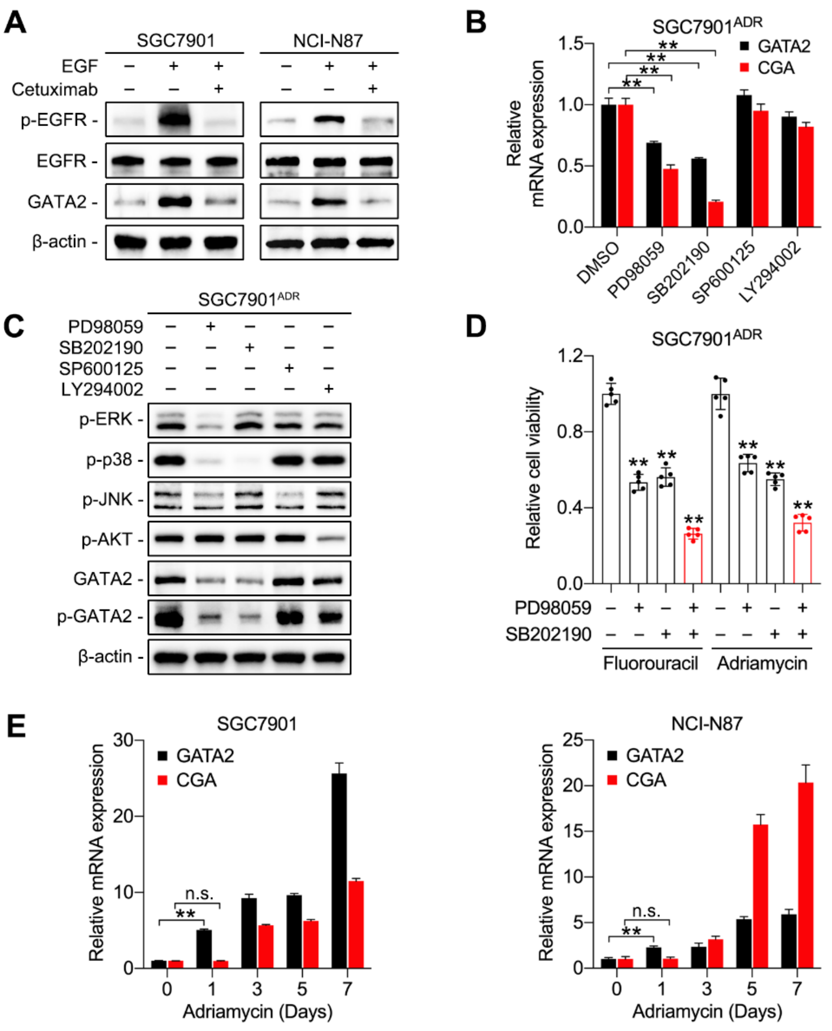

Fig. S8. The reciprocal positive regulation between GATA2 and CGA-EGFR signaling, related to Fig. 5.

(A) Immunoblotting of p-EGFR, EGFR and GATA2 in SGC7901 and NCI-N87 cells pretreated with cetuximab followed by EGF (50 ng/ml) treatment. **(B)** RT-qPCR analyses of GATA2 and CGA expression in SGC7901^{ADR} cells treated with the indicated compounds. **(C)** Immunoblotting of GATA2 and p-GATA2 (Ser192) in SGC7901^{ADR} cells treated with the indicated compounds. **(D)** Viability of SGC7901^{ADR} cells treated with PD98059 and/or

SB202190 in the presence of chemotherapy. **(E)** RT-qPCR analyses of GATA2 and CGA expression in SGC7901 and NCI-N87 cells treated with low concentrations of adriamycin (0.5 μg/ml) for the indicated time periods. Data are presented as mean ± SEM. $^{**}p < 0.01$ by one-way ANOVA followed by Dunnett's multiple comparison in (B and D) or by Student's *t* test in (E).

Figure S9

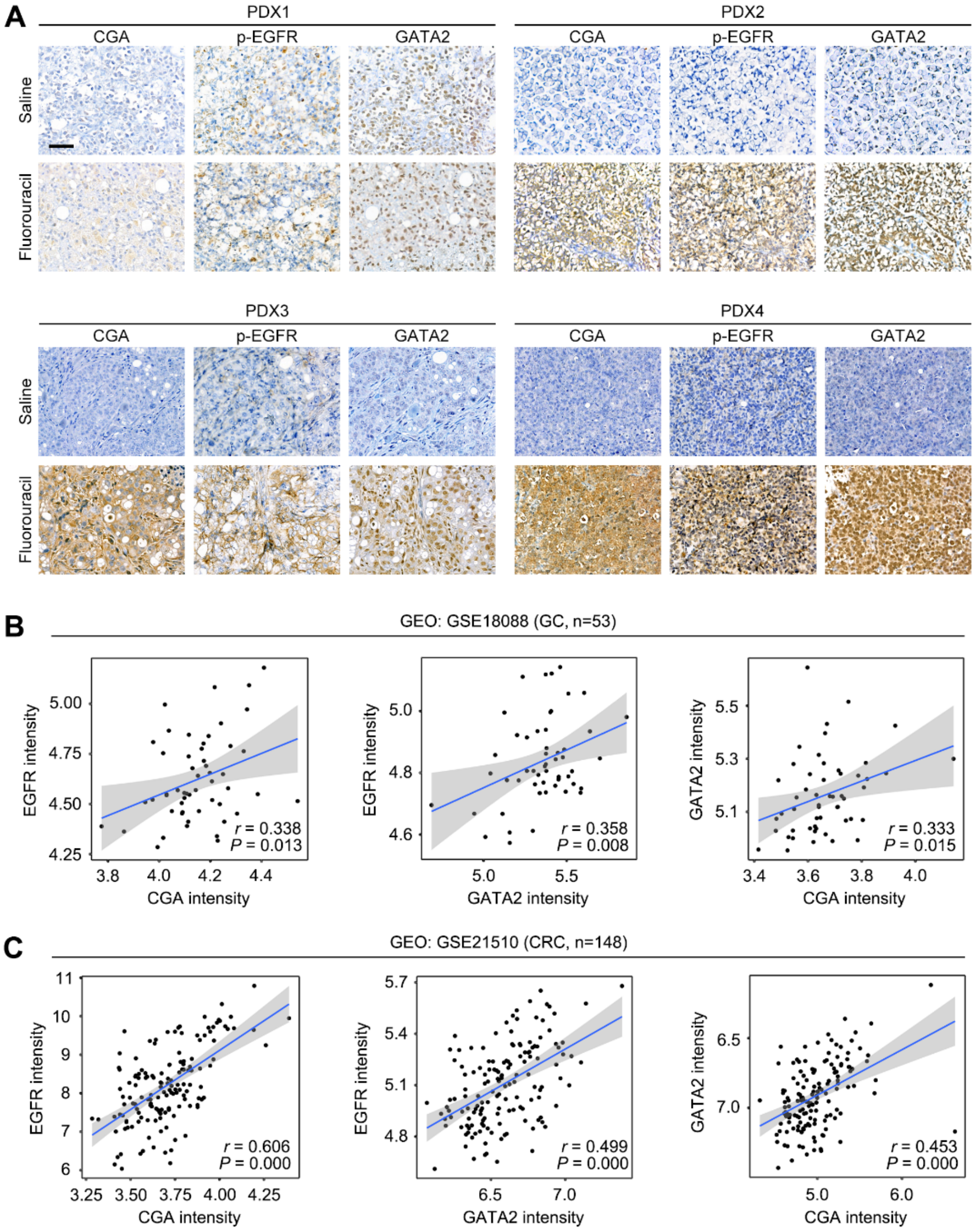

Fig. S9. Elevated CGA and GATA2 expression levels in GC patients who received chemotherapy, related to Fig 6.

(A) IHC staining of CGA, p-EGFR and GATA2 in four PDXs treated with saline control or fluorouracil (scale bar, 50 μm). The CGA images are the same as shown in Figure 1E.

(B, C) Scatter plots showing correlated expression of CGA, GATA2 and EGFR mRNA in

a GC (B) and a colorectal cancer (CRC, C) dataset of GEO. Pearson correlation coefficients (*r*) and p values are shown.

Figure S10

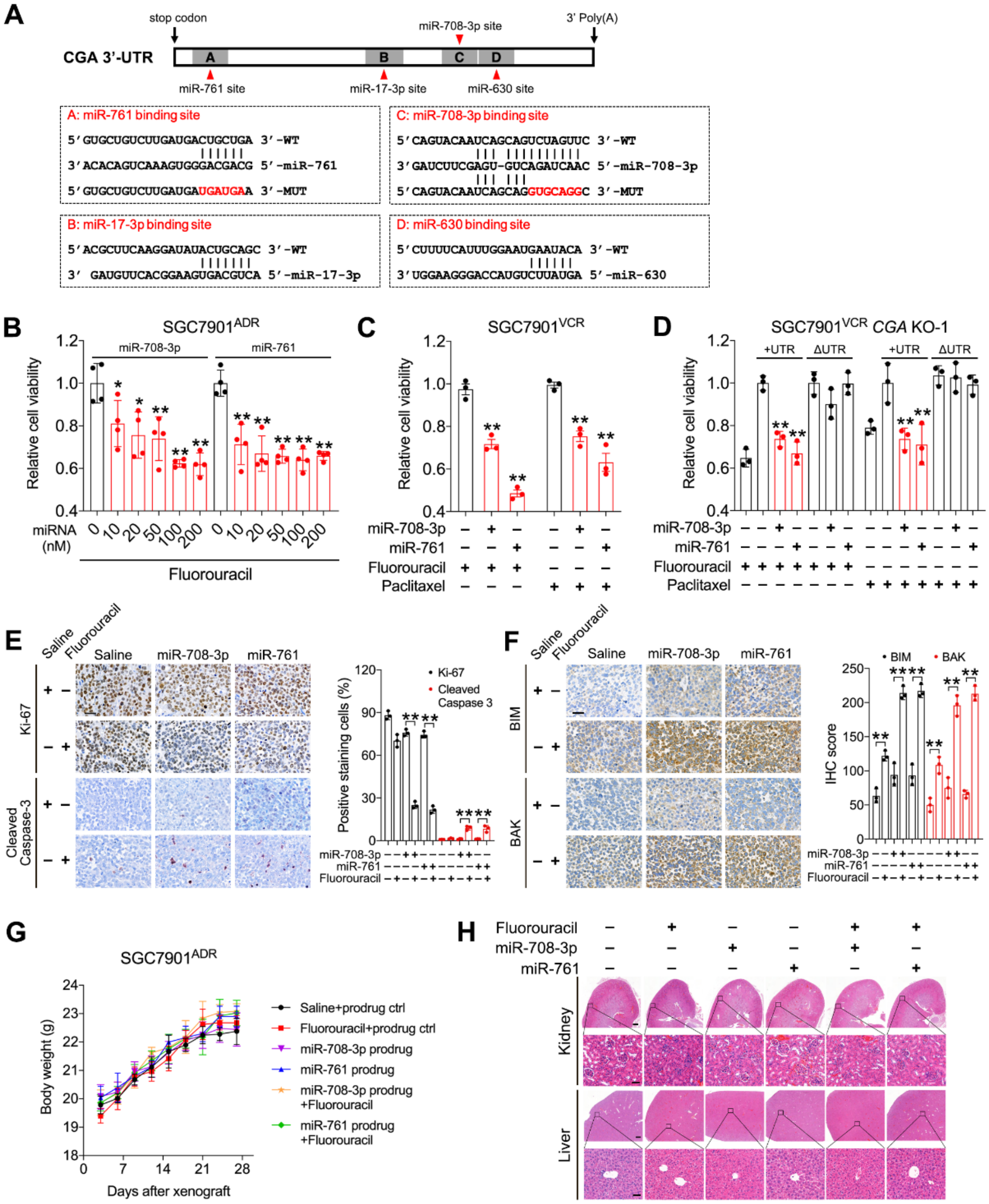

Fig. S10. miR-708-3p and miR-761 sensitize chemoresistant GC cells by targeting CGA, related to Fig. 7.

(A) Predicted duplex sequences between the WT or MUT 3'-UTR of CGA and the indicated miRNAs. The red portions of the sequences represent the MUT miRNA binding

sites in the 3'-UTR of CGA. **(B, C)** Viability of $SGC7901^{ADR}$ (B) and $SGC7901^{VCR}$ (C) cells transfected with miR-708-3p or miR-761 at the indicated concentrations in the presence of chemotherapy. **(D)** Viability of $CGA^{-/-}$ $SGC7901^{VCR}$ cells transfected with the indicated CGA vectors in combination with miR-708-3p or miR-761 in the presence of chemotherapy. **(E, F)** Representative IHC staining of Ki-67 and cleaved Caspase-3 (E) and BIM and BAK (F) in tumors harvested from the indicated groups (scale bar, 50 μm). The percentages of Ki-67- and cleaved Caspase-3-positive cells and the levels of BIM and BAK protein expression were measured. **(G)** Records of mouse body weights after the indicated treatments. n=7 mice for the saline, fluorouracil and intratumorally injected miRNA prodrugs; 8 mice for fluorouracil in combination with miRNA prodrugs. Data are presented as mean ± SEM. *p < 0.05, **p < 0.01 by one-way ANOVA followed by Dunnett's multiple comparison in (B-D) or by Student's *t* test in (E and F).

Table S1. Secreted proteins identified in the conditioned medium from SGC7901, SGC7901^{VCR} and SGC7901^{ADR} cells.

Cell line	Protein name
SGC7901	ANXA2, ANXA5, B2M, CALM1, CD59, CDA, CFD, CLIC1, CLU, CMBL, COL5A1, CST3, CTGF, CTSS, CYR61, ECH1, EIF6, FSTL3, GLO1, GLOD4, GSTO1, GSTP1, HBB, HIST1H4A, HLA-A, HNRNPA2B1, HRNR, HSPB1, HSPE1, HSPG2, IFI30, IGFBP1, IGFBP4, IGFBP6, IGFBP7, IL18, KRT1, KRT2, KRT5, KRT9, LCN2, LDHA, LDHB, LGALS1, LGALS3, LGALS3BP, LMAN2, MDH1, MDH2, METRNL, MFAP5, MIF, MSLN, NME2, PCNA, PFN1, PGAM1, PGLS, PI3, PNP, PPA1, PPIA, PPIB, PPIC, PRDX1, PRDX3, PRDX4, PRDX6, PSMA1, PSMA2, PSMA3, PSMA4, PSMA5, PSMA6, PSMA7, PSMB1, PSMB4, PSMB5, PSMB6, PSMB8, RPS27A, S100A4, SRM, TAGLN2, TIMP1, TIMP2, TPI1, TPM3, TPM4, TXN, TXNDC17, WISP2, YWHAB, YWHAE, YWHAG, YWHAZ, C19orf10, APOA1BP, GNB2L1, DCI
SGC7901^{VCR}	ABHD14B, ACP1, ACTG1, ADAM22, AK2, AMBP, ANXA1, ANXA2, ANXA5, ARHGDIA, ARPC2, ARPC4, B2M, BLVRB, BOLA2, C1QBP, C3, CALM1, CAPZB, CBR1, CDA, CFD, CFL1, CGA, CLIC1, CLIC4, CLU, CMBL, CMPK1, CNBP, CNPY2, COL5A1, COTL1, CRIP1, CST3, CSTB, CTGF, CTSS, CTSZ, CTTN, CUTA, CYCS, CYR61, DAG1, DDT, DSTN, DUT, DYNLL2, ECH1, ECHS1, EEF1D, EFEMP1, EIF6, ENOPH1, ERH, ERP29, ESD, FAM49B, FDX1, FHL2, FHL3, FKBP1A, FSTL3, GAPDH, GCSH, GGCT, GLO1, GLOD4, GNPDA1, GRB2, GSTM1, GSTM3, GSTO1, GSTP1, HADH, HBB, HEXB, HIBADH, HINT1, HIST1H4A, HIST1H4G, HIST2H3A, HLA-A, HLA-B, HNRNPA2B1, HNRNPAB, HNRNPC, HNRNPH2, HPRT1, HRNR, HSD17B10, HSPA5, HSPB1, HSPE1, HSPG2, IFI30, IGFBP1, IGFBP6, IGFBP7, IL6, ISOC1, KIAA0100, KRT1, KRT17, KRT18, KRT2, KRT5, KRT6B, KRT9, LASP1, LDHA, LDHB, LGALS1, LGALS3, LGALS3BP, LMAN2, LMNA, LSM3, LTF, MDH1, MDH2, MFAP2, MIF, MSLN, MYL12A, MYL6, NIT1, NIT2, NME2, NPC2, NQO1, NQO2, NUDT5, OTUB1, PAFAH1B2, PARK7, PCBD1, PCNA, PDCD6, PEBP1, PFN1, PGAM1, PGK1, PGLS, PI3, PNP, PPA1, PPIA, PPIB, PPIF, PPIH, PPP4C, PRDX1, PRDX2, PRDX3, PRDX4, PRDX6, PROCR, PSMA1, PSMA2, PSMA3, PSMA4, PSMA5, PSMA6, PSMA7, PSMB1, PSMB2, PSMB3, PSMB4, PSMB5, PSMB6, PSMB7, PSMB8, PSMB9, PSMD9, PSME1, PSME2, PSPH, PTMA, RAB1B, RAN, RBP4, RPS12, RPS27A, RPS3, S100A4, S100A6, SFN, SLPI, SNRPD1, SNRPD3, SOD1, SOD2, SRI, SRM, STC2, SUMF2, TAGLN2, TBCA, TGFB2, TIMP1, TIMP2, TKT, TMSB10, TPI1, TPM3, TPM4, TPT1, TRIP6, TSN, TSNAX, TWSG1, TXN, TXNDC17, UBE2I, UBE2M, UBE2V1, UCHL3, UFC1, VPS29, WISP2, YWHAB, YWHAE, YWHAG, YWHAQ, YWHAZ, C19orf10, APOA1BP, GNB2L1, DCI, C1orf128, THOC4, TCEB1, TUBB2C, RCL

SGC7901^{ADR}	ABHD14B, ACP1, ACTG1, ADAM22, AHSG, AK1, AK2, AKR1A1, AKR1B1, ALDOA, ANP32A, ANXA1, ANXA2, ANXA3, ANXA5, APEX1, APOA1, APRT, ARCN1, ARF1, ARHGDIA, ARPC2, ARPC3, ARPC4, ARPC5, ATP6AP2, B2M, BLVRB, BOLA2, C1QTNF3, C3, C4B, CALM1, CAPZB, CBR1, CBX1, CD59, CDKN2A, CFD, CFL1, CFL2, CGA, CHAC2, CLEC3B, CLIC1, CLU, CMBL, CMPK1, CNBP, CNPY2, COL1A1, COL1A2, COL3A1, COL5A1, COPS6, COTL1, COX6B1, CPPED1, CREG1, CRIP1, CRYZ, CSRP1, CST3, CSTB, CTGF, CTSZ, CUTA, CYCS, CYR61, DAG1, DBI, DCTPP1, DDT, DHX9, DSTN, DUT, DYNLL2, ECH1, ECHS1, EEF1D, EFEMP1, EIF3G, EIF3J, EIF6, ENO1, ERH, ERP29, ESD, EXOSC2, F5, FABP3, FBN1, FDPS, FHL2, FKBP1A, FKBP2, FSTL3, GAPDH, GDF15, GGCT, GGH, GLO1, GLOD4, GRB2, GSTM1, GSTM3, GSTO1, GSTP1, H2AFJ, HADH, HBA1, HBB, HIST1H2BL, HIST1H4A, HLA-A, HNRNPA1, HNRNPA2B1, HNRNPAB, HNRNPC, HNRNPH1, HNRNPH3, HPRT1, HSD17B10, HSD17B4, HSP90AB1, HSPB1, HSPE1, HSPG2, IAH1, IFI30, IGF2, IGFBP1, IGFBP2, IGFBP4, IGFBP6, IGFBP7, IL6, ISG15, KRT1, KRT10, KRT18, KRT2, KRT8, KRT9, LDHA, LDHB, LGALS1, LGALS3, LMAN2, LMNA, LSM3, LTF, MAGOH, MAGOHB, MDH1, MDH2, METRN, MIF, MSLN, MTAP, MYL12A, MYL6, NEDD8, NIT2, NME1, NME2, NPM1, NQO2, NT5C, NUDT21, NUDT5, NUTF2, OAF, OTUB1, PAFAH1B2, PAFAH1B3, PARK7, PCBD1, PCMT1, PCNA, PCSK9, PDCD5, PDXK, PEBP1, PFDN2, PFN1, PGAM1, PGAM4, PGLS, PGP, PI3, PNP, PNPO, POLR2H, PPA1, PPIA, PPIB, PPP1CC, PPP2CA, PRDX1, PRDX2, PRDX3, PRDX4, PRDX6, PROCR, PSMA1, PSMA2, PSMA3, PSMA4, PSMA5, PSMA6, PSMA7, PSMB1, PSMB2, PSMB3, PSMB4, PSMB5, PSMB6, PSMB7, PSMB8, PSMB9, PSME1, PSME2, PSPH, PXDN, RAB10, RAB11B, RAB1B, RAB7A, RAB8A, RAC1, RAN, RBM8A, RBP4, RNASE4, RPS11, RPS12, RPS18, RPS20, RPS21, RPS27A, RPS28, RPS3, RPS4X, RPSA, S100A11, S100A4, S100A6, SFN, SLPI, SNRPD1, SNRPD3, SOD1, SOD2, SPON2, SPP2, SRI, SRM, SRP9, SSBP1, STC2, STMN1, SUMO4, TAGLN2, TBCA, TGFB1, TGFB2, THBS1, TIMP1, TIMP2, TNFRSF12A, TPI1, TPM3, TPM4, TPT1, TRIP6, TRMT112, TSN, TUBA1C, TWSG1, TXN, TXNDC17, UBE2I, UBE2M, UBE2V1, UBE2V2, UCHL3, UFC1, VPS29, WISP2, YWHAB, YWHAE, YWHAG, YWHAQ, YWHAZ, ZBTB8OS, C19orf10, APOA1BP, GNB2L1, THOC4, TCEB1, RCL, C14orf166, CXorf26, SNRPEL1, C11orf73, PLEC1, CTSL1

Table S2. Clinicopathological characteristics of GC patients with paired specimens before and after neoadjuvant chemotherapy.

No.[1]	Gender	Age	Histology	Differentiation	TNM stage	Chemotherapy regimen[2]	Response to chemotherapy[3]
1	male	44	adenocarcinoma	moderate	T2N0Mx	FOLFOX	SD
2	male	50	adenocarcinoma	moderate	T3N0	FOLFOX	SD
3	female	59	adenocarcinoma	poor	T4aN3a	FOLFOX	SD
4	male	66	adenocarcinoma	moderate&poor	pT3N3	FOLFOX	SD
5	male	66	adenocarcinoma	moderate	pT3N1	DCF	PR
6	male	65	adenocarcinoma	poor	pT3N3M0	FOLFOX	PD
7	male	60	adenocarcinoma	moderate&poor	pT4N2M0	FOLFOX	PD
8	male	44	adenocarcinoma	poor	T4bN0	FOLFOX	SD
9	male	53	adenocarcinoma	moderate	T4aN2	ECF	SD
10	male	66	adenocarcinoma	well&moderate	T3N1	FOLFOX	SD
11	male	68	adenocarcinoma	poor	T4aN3b	DCF	SD
12	male	58	adenocarcinoma	poor	T3N1	ECF	SD
13	male	57	adenocarcinoma	moderate	rT3N3b	FOLFOX	SD
14	male	67	adenocarcinoma	poor	T3N3a	FOLFOX	SD
15	male	56	adenocarcinoma	poor	T3N3a	XELOX	SD
16	male	48	adenocarcinoma	moderate	T3N0	FOLFOX	SD
17	female	62	adenocarcinoma	moderate	T4aN3a	DCF	SD
18	male	58	adenocarcinoma	poor	T4aN1	FOLFOX	SD
19	female	50	adenocarcinoma	poor	T4aN3a	SOX	SD
20	male	69	adenocarcinoma	poor	TXN0	EOX	PR
21	female	57	adenocarcinoma	well&moderate	T3N3a	FOLFOX	SD
22	male	67	adenocarcinoma	poor	T4aN3a	DCF	PR
23	male	51	adenocarcinoma	poor	T3N2	DCF	SD
24	female	58	adenocarcinoma	poor	T3N0	SOX	SD

25	male	36	adenocarcinoma	moderate&poor	T3N0	DCF	PR
26	male	35	adenocarcinoma	moderate&poor	T4bN2	DCF	SD
27	male	63	adenocarcinoma	moderate	T4bN2	SOX	PD
28	male	52	adenocarcinoma	moderate&poor	T4aN2	SOX	SD
29	male	55	adenocarcinoma	moderate&poor	T4aN2	SOX	SD
30	female	55	adenocarcinoma	moderate&poor	T1bN3a	DOX	PR
31	male	54	adenocarcinoma	poor	T4N1	DCF	SD
32	male	59	adenocarcinoma	well&moderate	T3N0	DCF	PR
33	female	48	adenocarcinoma	moderate	pT4N3M0	SOX	PD
34	male	80	adenocarcinoma	poor	pT0N1M0	SOX	PD
35	male	47	adenocarcinoma	moderate&poor	pT2N1M0	FOLFIRI	PD
36	female	47	adenocarcinoma	moderate&poor	pT1bN1M0	FOLT	PD
37	female	55	adenocarcinoma	poor	pT4N1M1	FOLT	SD

1. Patients 1, 2, 3, 4, 5 and 6 in Fig. 1D and Fig. 6A denote No. 12, 13, 29, 33, 35 and 6 in this table, respectively. Patients 1, 2, 3, 4, 5 and 6 in fig. S1D denote No. 5, 20, 22, 25, 30 and 32 in this table, respectively.

2. Chemotherapy regimens: FOLFOX (fluorouracil, leucovorin and oxaliplatin); EOX (epirubicin, oxaliplatin and capecitabine); DCF (docetaxel, cis-platinum and fluorouracil), ECF (epirubicin, cis-platinum and fluorouracil); XELOX (capecitabine and oxaliplatin); SOX (S-1 and oxaliplatin); FOLFIRI (fluorouracil, leucovorin and irinotecan); FOLT (fluorouracil, leucovorin, oxaliplatin and docetaxel).

3. PR, partial response; SD, stable disease; PD, progressive disease.

Table S3. Clinical information of GC tissues used to generate the PDX model.

PDX No.	Gender	Age	Histology	Differentiation	AJCC stage	PDX passage
1	male	47	adenocarcinoma	poor&moderate	2	P2
2	male	68	adenocarcinoma	poor	4	P2
3	male	66	adenocarcinoma	moderate	4	P3
4	female	55	adenocarcinoma	poor	4	P4

Table S4. Docking analysis of CGA and the ECD of EGFR by using the ClusPro server.

Residues in CGA	Residues in the ECD of EGFR
K44.HZ3	Y88.OH
R42.HH12	N86.OD1
R42.HH22	N86.OD1
Y37.OH	S262.H
Y37.HH	G281.O
Y37.H	C240.O
T54.O	T239.HG1
S55.HG	T239.OG1
S55.OG	T239.H
K91.HZ1	D238.OD1
Y89.HH	Q193.O
D6.OD2	W584.H
Q5.OE1	E578.H
S85.HG	E221.OE2
M29.O	N599.HD22
Q13.HE22	N599.OD1
S34.H	N580.OD1
D3.OD1	T249.H
D3.OD1	T249.HG1

Table S5. The expression levels of eight transcription factors in SGC7901, SGC7901^{ADR} and SGC7901^{VCR} cells.

Gene	Log2 Ratio (SGC7901^{ADR}/SGC7901)	*P* value	FDR	Log2 Ratio (SGC7901^{VCR}/SGC7901)	*P* value	FDR
CEBPA	2.921111225	0.000000	0.000000	2.710206444	0.000000	0.000000
EGR1	3.049297067	0.000000	0.000000	3.257048388	0.000000	0.000000
FOXD1	1.490615045	0.000000	0.000000	1.753723252	0.000000	0.000000
GATA2	1.690832402	0.000000	0.000000	1.64289388	0.000000	0.000000
GATA3	6.033496871	0.000031	0.000053	10.34487385	0.000000	0.000000
JUN	1.322488813	0.000000	0.000000	1.496223127	0.000000	0.000000
NFIL3	1.639760573	0.000000	0.000000	1.675893783	0.000000	0.000000
RUNX2	7.061719798	0.000000	0.000000	7.508497903	0.000000	0.000000

Data came from Chen. et al, *Cancer Lett*, 2014.

Table S6. Clinical information of GC patients who did not receive treatment.

No.	Gender	Age	Histology	TNM stage
1	male	63	adenocarcinoma	T4aN0M0
2	female	64	adenocarcinoma	cT4aN3aM0
3	male	43	adenocarcinoma	T3N3aM0
4	male	66	adenocarcinoma	cT4aN0M0
5	male	49	adenocarcinoma	cT4aN3aM0
6	male	52	adenocarcinoma	cT4aN3aM0
7	female	43	adenocarcinoma	cT4aN3aM0
8	male	43	adenocarcinoma	T3N3aM0
9	male	82	adenocarcinoma	cT4aN3aM0
10	male	62	adenocarcinoma	T3N1M0
11	male	62	adenocarcinoma	T4aN3M0
12	male	72	adenocarcinoma	T3N3aM0
13	male	65	adenocarcinoma	T4aN3bM0
14	male	58	adenocarcinoma	T3N2M0
15	male	58	adenocarcinoma	T4aN1M0
16	male	59	adenocarcinoma	T4aN3a
17	male	77	adenocarcinoma	T4aN3bM0
18	male	62	adenocarcinoma	T4aN2M0
19	male	74	adenocarcinoma	T3N3M0
20	male	66	adenocarcinoma	T4aNxM0
21	male	72	adenocarcinoma	T4aN3b
22	male	63	adenocarcinoma	T4bN2M1
23	male	59	adenocarcinoma	T4aN3aM0
24	male	52	adenocarcinoma	T4aN3a
25	male	71	adenocarcinoma	T3N1
26	male	64	adenocarcinoma	T3N3M0
27	male	72	adenocarcinoma	T4aN1
28	male	50	adenocarcinoma	T3N2M0
29	male	50	adenocarcinoma	T3N3aM0
30	male	58	adenocarcinoma	T3N3aM0
31	male	70	adenocarcinoma	T3N3b
32	male	52	adenocarcinoma	T2Nx
33	female	59	adenocarcinoma	T2Nx
34	male	49	adenocarcinoma	T1bNx
35	male	66	adenocarcinoma	T2Nx
36	female	55	adenocarcinoma	T2a1N0
37	female	70	adenocarcinoma	T2Nx
38	male	50	adenocarcinoma	T3N0M0

39	female	47	adenocarcinoma	T1bN1M0
40	female	53	adenocarcinoma	T1Nx
41	male	68	adenocarcinoma	T2N0M0
42	female	56	adenocarcinoma	T2Nx

Table S7. Clinicopathological characteristics of GC patients who provided plasma samples and received neoadjuvant chemotherapy.

No.[1]	Gender	Age	Differentiation	TNM stage	Chemotherapy regimen[2]	Response to chemotherapy[3]	Survival time (month)	Status
1	male	50	moderate	T3N0	FOLFOX	SD	55	dead
2	male	69	poor	T3N1	FOLFOX	SD	23	dead
3	female	46	poor	T4aN3a	FOLFOX	SD	7	dead
4	male	66	moderate	T3N1	FOLFOX	PR	84	censored
5	male	58	moderate	T3N1	ECF	SD	2	dead
6	male	61	poor	T4aN3a	FOLFOX	PR	17	dead
7	male	57	moderate	rT3N3b	FOLFOX	SD	8	censored
8	male	67	poor	T3N3a	FOLFOX	SD	5	dead
9	male	65	poor	T4aN1	ECF	PR	50	censored
10	male	40	moderate	T1bN2	SOX	PR	72	censored
11	male	38	poor	T4aN1	SOX	SD	4	dead
12	female	62	moderate	T4aN3a	DCF	SD	24	dead
13	female	58	poor	T4aN1	FOLFOX	SD	20	dead
14	female	50	poor	T4aN3a	SOX	SD	7	dead
15	male	56	moderate	T1aN0	DS	-	39	censored
16	female	57	moderate	T3N3a	FOLFOX	SD	17	dead
17	male	47	moderate	T4aN0	DCF	SD	38	censored
18	male	67	poor	T4aN3a	DCF	SD	66	censored
19	male	51	poor	T3N2	DCF	PR	62	censored
20	male	48	moderate	T2N1	DCF	SD	59	censored
21	male	67	poor	T3N1	FOLFOX	PR	57	censored
22	female	46	poor	T3N0	DCF	SD	40	censored
23	female	43	poor	T4bN3a	DCF	SD	28	censored

24	male	44	poor	T3N0	SOX	SD	17	dead
25	female	58	poor	T3N0	SOX	SD	52	censored
26	male	35	moderate&poor	T4bN2	DCF	SD	4	dead
27	male	56	poor	T4aN2	DF	SD	25	censored
28	male	63	moderate	T3N3a	SOX	SD	8	dead
29	male	52	moderate&poor	T4aN2	SOX	SD	50	censored
30	female	63	poor	T3N2	DOX	SD	18	censored
31	male	62	moderate	T3N1	DCF	SD	10	censored
32	male	55	moderate&poor	T4aN2	SOX	SD	51	censored
33	male	65	poor	T4bN3	DCF	SD	1	dead
34	male	68	moderate	T4aN3a	DCF	SD	10	censored
35	male	39	moderate&poor	T4aN3b	DCF	SD	13	censored
36	female	58	poor	T4aN3b	DCF	SD	5	dead
37	female	55	moderate&poor	T1bN3a	DOX	PR	26	censored
38	male	59	moderate	T3N0	DCF	SD	50	censored
39	male	66	moderate&poor	T4aN0	XELOX	PR	48	censored
40	male	57	well&moderate	T3N1	S-1	PR	51	censored
41	female	29	poor	T4N0	-	SD	13	censored

1. The pre- and post-operative plasma CGA levels of No. 6, 8, 10, 13, 14, 15, 17, 22, 24, 27, 28, 36, 39, 40 and 41 are shown in Fig. 6D.

2. Chemotherapy regimens: DS (docetaxel and S-1); DF (cis-platinum and fluorouracil); DOX (docetaxel, oxaliplatin and capecitabine). Patient 41 was transferred from a nonlocal hospital and her chemotherapy treatment history is not available.

3. PR, partial response; SD, stable disease. The information of response to chemotherapy is not available for No. 15.

Table S8. Clinicopathological characteristics of GC patients who provided plasma samples and received palliative chemotherapy.

No.	Gender	Age	AJCC stage	Chemotherapy regimen[1]	Response to chemotherapy[2]	Survival time (month)[3]	Status
1	female	19	4	DCF	SD	2.4	censored
2	male	67	4	SOX	PD	11	dead
3	male	66	4	FLOT	SD	-	-
4	male	75	4	SOX	SD	14.6	dead
5	male	56	4	FLOT	SD	16.6	dead
6	male	57	4	DCF	PD	-	-
7	male	61	4	SOX	PD	11.5	censored
8	female	66	4	SP	PD	-	-
9	female	35	4	DCF	PD	-	-
10	male	75	4	SOX	PD	21.1	dead
11	male	54	4	SOX	PD	21.8	dead
12	male	70	4	DCF	SD	7.5	dead
13	male	50	4	DCF	SD	-	-
14	male	66	4	SOX	PD	5.4	censored
15	male	56	4	DCF	SD	7.9	dead
16	male	55	4	DCF	PD	-	-
17	male	52	4	DCF	SD	-	-
18	male	77	4	SOX	SD	-	-
19	male	54	4	DCF	PD	6	dead
20	male	53	4	XELOX	PD	-	-
21	male	66	4	SOX	SD	5	dead
22	male	80	4	SOX	PD	6	dead
23	male	65	4	DCF	PD	-	-

24	male	76	4	SOX	PD	-	-
25	male	59	4	DCF	PD	-	-
26	male	62	4	XELOX	SD	53.8	censored
27	male	75	4	FOLFOX	SD	-	-
28	male	76	4	XELOX	SD	-	-
29	female	51	4	XELIRI	PD	-	-
30	male	70	4	DS	SD	-	-
31	female	51	4	DCF	PD	-	-
32	female	47	4	DF	SD	15.4	censored
33	male	56	4	Fluorouracil	SD	-	-
34	male	57	4	DCF	SD	-	-
35	male	62	4	FOLFIRI	SD	-	-
36	male	65	4	XELOX	SD	-	-
37	male	75	4	XELOX	SD	-	-
38	male	75	3	DCF	PD	-	-
39	male	40	4	DCF	PD	-	-
40	male	56	4	DCF	PD	-	-
41	male	41	4	DCF	PD	3.3	dead
42	female	59	4	DCF	PD	-	-
43	male	60	4	EOX	SD	5.9	dead
44	male	30	4	DCF	SD	-	-
45	female	31	4	DOX	SD	5.7	censored
46	male	35	4	DCF	PD	0.2	dead
47	male	60	4	XELOX	PD	16.3	dead
48	female	64	4	FLOT	SD	-	-
49	male	59	4	EOX	SD	11.3	dead
50	male	75	4	SOX	SD	7.3	dead

51	male	51	3	EOX	PD	-	-
52	female	37	4	DCF	PD	-	-
53	male	61	4	DCF	PD	3.1	censored
54	female	55	4	FOLFOX	PD	-	-
55	male	61	4	DCF	PD	-	-
56	male	71	4	FOLFOX	PD	-	-

1. Chemotherapy regimens: SP (S-1 and cis-platinum); XELIRI (capecitabine and irinotecan); EOX (epirubicin, oxaliplatin and capecitabine).

2. SD, stable disease; PD, progressive disease.

3. Twenty-three patients received survival follow-up.

Table S9. miRNAs targeting CGA predicted by the overlap between miRWalk (version 2.0) and TargetScan (version 7.2).

miRNA	Gene symbol	Position in 3'-UTR of CGA	Context++ score
hsa-miR-7843-3p	CGA	121-127	84
hsa-miR-7706	CGA	120-126	89
hsa-miR-766-5p	CGA	151-157	96
hsa-miR-761	CGA	28-34	94
hsa-miR-708-3p	CGA	181-187	97
hsa-miR-6891-5p	CGA	153-160	99
hsa-miR-6876-5p	CGA	149-155	97
hsa-miR-6516-5p	CGA	226-233	99
hsa-miR-651-3p	CGA	96-103	99
hsa-miR-630	CGA	203-209	89
hsa-miR-6124	CGA	94-100	98
hsa-miR-525-5p	CGA	38-44	94
hsa-miR-5192	CGA	88-94	99
hsa-miR-501-5p	CGA	126-132	86
hsa-miR-4999-5p	CGA	205-212	99
hsa-miR-4742-3p	CGA	204-210	90
hsa-miR-449c-5p	CGA	145-151	99
hsa-miR-4499	CGA	178-184	98
hsa-miR-4476	CGA	149-155	96
hsa-miR-4463	CGA	178-184	96
hsa-miR-4428	CGA	90-96	98
hsa-miR-3714	CGA	146-152	98
hsa-miR-362-5p	CGA	125-132	99
hsa-miR-3619-5p	CGA	28-34	92
hsa-miR-34b-5p	CGA	145-151	99
hsa-miR-342-3p	CGA	77-83	94
hsa-miR-320c	CGA	138-145	99
hsa-miR-3180-5p	CGA	38-44	97
hsa-miR-3173-3p	CGA	153-160	99
hsa-miR-2682-5p	CGA	145-151	99
hsa-miR-214-3p	CGA	28-34	97
hsa-miR-17-3p	CGA	134-140	98
hsa-miR-1298-5p	CGA	199-205	97
hsa-miR-1285-5p	CGA	78-85	99
hsa-miR-127-5p	CGA	121-127	88
hsa-miR-1183	CGA	163-170	99

Table S10. Antibodies used in this study.

Antibodies	Source	Catalog #
Rabbit anti-human CGA	Abcam	ab92738
Mouse anti-human α-Tubulin	Sigma-Aldrich	T5168
Rabbit anti-human EGFR (for IB)	Abcam	06-847
Rabbit anti-human EGFR (for IP)	Proteintech	51071-2-AP
Mouse anti-human EGFR (for IF)	Abcam	ab30
Rabbit anti-human p-EGFR (Tyr1068)	Cell Signaling Technology	3777
Rabbit anti-human ERK	Cell Signaling Technology	4695
Rabbit anti-human p-ERK (Thr202/Tyr204)	Cell Signaling Technology	4370
Rabbit anti-human AKT	Cell Signaling Technology	4691
Rabbit anti-human p-AKT (Ser473)	Cell Signaling Technology	4060
Rabbit anti-human β-actin	Cell Signaling Technology	4970
Rabbit anti-Flag	Sigma-Aldrich	F7425
Rabbit anti-human Ki-67	Abcam	ab15580
Rabbit anti-human cleaved Caspase-3	Abcam	ab2302
Rabbit anti-human CGB	Proteintech	11615-1-AP
Mouse anti-human FSHB	RD Systems	MAB4310
Sheep anti-human LHB	RD Systems	AF8016
Mouse anti-human TSHB	RD Systems	MAB57941
Rabbit anti-human GATA2	Cell Signaling Technology	4595
anti-human p-GATA2 (Ser192)	Thermo Fisher Scientific	PA5-105538
anti-human p-p38 (Thr180/Tyr182)	Cell Signaling Technology	4511
anti-human p-JNK (Thr183/Tyr185)	Cell Signaling Technology	4668
Donkey polyclonal anti-Rabbit IgG, HRP conjugated	GE Healthcare Life Sciences	NA934
Sheep polyclonal anti-Mouse IgG, HRP conjugated	GE Healthcare Life Sciences	NA931
Donkey anti-Mouse IgG (H+L) Highly Cross-Adsorbed, Alexa Fluor 488 conjugated	Thermo Fisher Scientific	A32766
Donkey anti-Rabbit IgG (H+L) Highly Cross-Adsorbed, Alexa Fluor Plus 594 conjugated	Thermo Fisher Scientific	A32754
Rabbit IgG	Proteintech	B900610

Rabbit anti-human BIM	Cell Signaling Technology	2933
Rabbit anti-human BAK	Cell Signaling Technology	12105
Mouse anti- human EEA1	Abcam	ab75852
Mouse anti- human CathD	Santa Cruz Biotechnology	sc377299
Donkey anti-Mouse IgG (H+L), Alexa Fluor 405 conjugated	Abcam	Ab175658
Rabbit anti-human EGFR, Alexa Fluor 488 conjugated	Cell Signaling Technology	5616

Table S11. Oligonucleotides used in this study.

Oligonucleotide	Sequence
qPCR primers for CGA	F: GCCCTGAACACATCCTGCAA<br>R: GCCCTGAACACATCCTGCAA
qPCR primers for GATA2	F: CAAGGCTCGTTCCTGTTCAG<br>R: TGCCCATTCATCTTGTGGTAG
qPCR primers for GAPDH	F: GCACCGTCAAGGCTGAGAAC<br>R: TGGTGAAGACGCCAGTGGA
qPCR primers for U6	F: TGGAACGCTTCACGAATTTGCG<br>R: GGAACGATACAGAGAAGATTAGC
qPCR primers for GBE1 of CGA promoter	F: CAACGTAAAAGGGCTGTACCT<br>R: AGCCATAGCCAGCAAGCTGG
qPCR primers for GBE2 of CGA promoter	F: ATGCATACTGGAAGGAAACA<br>R: TTTGCACACAGAGATTATGAC
qPCR primers for distal region of CGA promoter	F: CCAGCTCTGGGAAGAACTCTC<br>R: TTATGAATGAGCCATTAGCC
qPCR primers for −77 kb of GATA2 locus	F: TGTCTACTCCTACAACAAGATG<br>R: CTTTAATGGCCACAGGATAC
qPCR primers for −3.9 kb of GATA2 locus	F: TTGACCTGCCTGGAGATGAG<br>R: CCCATGGCGACGCAGATAG
qPCR primers for −3.0 kb of GATA2 locus	F: GCAGAGATAAGGAACACCATT<br>R: GAAGATAATGAATAGCCAGTCG
qPCR primers for −1.8 kb of GATA2 locus	F: AGAGCAGTTATTCAGCATGG<br>R: ACCATCCAGACCTTCCTTAAC
qPCR primers for +9.5 kb of GATA2 locus	F: TGTTTTCTCTCACGGCATCT<br>R: GACAAAGCCTTGCCCTTATG
qPCR primers for +14.5 kb of GATA2 locus	F: AGTGACAAAAATCCAGCCAC<br>R: CATGCAGCTTATCAAAAGCA
Biotin-labeled probe for GBE1 of CGA promoter	F: AATCATTCAGATAAAAAAGAAATAAA<br>R: TTTATTTCTTTTTTATCTGAATGATT
Competitor probe for GBE1 of CGA promoter	F: AATCATTCAGATAAAAAAGAAATAAA<br>R: TTTATTTCTTTTTTATCTGAATGATT
Mutant probe for GBE1 of CGA promoter	F: AATCATGAGAGCGGGGGAGAATAAA<br>R: TTTATTCTCCCCCGCTCTCATGATT
Biotin-labeled probe for GBE2 of CGA promoter	F: AAACAAATTTGTTTATCACAGAAAAT<br>R: ATTTTCTGTGATAAACAAATTTGTTT

Competitor probe for GBE2 of CGA promoter	F: AAACAAATTTGTTTATCACAGAAAAT<br>R: ATTTTCTGTGATAAACAAATTTGTTT
Mutant probe for GBE2 of CGA promoter	F: AAACAAAGCCCACCCGCTGTGGAAAAT<br>R: ATTTTCCACAGCGGGTGGGCTTTGTTT
scrambled siRNA control	UUCUCCGAACGUGUCACGUTT
siRNA#1 targeting CGA	GCAGCUAUCUUUCUGGUCACA
siRNA#2 targeting CGA	GAUUACAGAUUGCCCAGAAUG
siRNA#1 targeting CGB	ACCGUCAACACCACCAUCUTT
siRNA#2 targeting CGB	ACAUGUCUCUCUCUUAGCGTT
siRNA#1 targeting LHB	ACCGUCAACACCACCAUCUTT
siRNA#2 targeting LHB	ACAUGUCUCUCUCUUAGCGTT
siRNA#1 targeting FSHB	GGAAAGCAAUCUGCUGCAATT
siRNA#2 targeting FSHB	GAAUGUCGUUUCUGCAUAATT
siRNA#1 targeting TSHB	GCUUAUUGCCUAACCAUCATT
siRNA#2 targeting TSHB	CCUGUUGCUUUAAGCUGUATT
siRNA#1 targeting GATA2	ACUACAGCAGCGGACUCUUTT
siRNA#2 targeting GATA2	CCUGUGGCCUCUACCACAATT
